# *Limosilactobacillus mucosae* LM410 Enhances Intestinal Barrier and Antioxidant Capacity in Weaned Piglets via Microbial Tyrosine Metabolism and AhR/STAT3 Pathway

**DOI:** 10.3390/antiox15101264

**Published:** 2026-10-01

**Authors:** Jing Liu, Rui Jia, Pengfei Wang, Yuhao Liang, Fen Feng, Fei Wang, Qiaoli Yang

**Affiliations:** 1 College of Animal Science and Technology, Gansu Agricultural University, Lanzhou 730070, China; 18294186456@163.com (J.L.);; 2 Gansu Province Modern Pig Engineering Technology Innovation Center, Lanzhou 730070, China

**Keywords:** *Limosilactobacillus mucosae*, weaned piglets, oxidative stress, gut microbiota, intestinal stem cell

## Abstract

Oxidative stress compromises intestinal barrier function and antioxidant defense in weaned piglets, causing substantial economic losses in the pig industry. Probiotic interventions to improve gut health are considered a highly promising solution; research indicates that certain Lactobacillus species can promote the proliferation of Lgr5^+^ stem cells and activate the AhR/STAT3 pathway by modulating microbiota metabolism, thereby enhancing intestinal barrier and antioxidant function. Although *Limosilactobacillus mucosae* (*L. mucosae*) has demonstrated antioxidant properties in rodent models, its involvement in modulating intestinal stem cell (ISC) fate in piglets, as well as the associated mechanisms, remains unclear. We hypothesized that *L. mucosae* could alleviate oxidative stress in weaned piglets by modulating intestinal microbiota and activating the aryl hydrocarbon receptor/signal transducer and activator of transcription 3 (AhR/STAT3) signaling-mediated intestinal epithelial renewal. We compared the gut microbiota composition between Hezuo pigs (a subgroup of the plateau-type Tibetan breed) and Landrace × Yorkshire (LY) pigs, and isolated *L. mucosae* LM410 from Hezuo pigs. The strain was evaluated for acid and bile tolerance, antioxidant activity, and inhibitory properties. Subsequently, a 14-day animal trial was conducted using Duroc × Landrace × Yorkshire (DLY) weaned piglets orally administered with 1 × 10^9^ CFU/mL *L. mucosae* LM410. Intestinal morphology, barrier integrity, immune responses, antioxidant capacity and cecal microbiota composition and metabolic pathways were assessed. We found that Hezuo pigs harbored a higher abundance of *L*. *mucosae* in the gut compared with LY pigs. The isolate *L. mucosae* LM410 from Hezuo pigs, demonstrated strong acid and bile salt tolerance, as well as antioxidant and antimicrobial activities, and effectively improved intestinal morphology, barrier integrity, immune responses, and antioxidant capacity in DLY weaned piglets. Mechanistically, *L. mucosae* LM410 reshaped the cecal microbiota by enriching short-chain fatty acid (SCFA)-producing bacteria and upregulating the tyrosine metabolic pathway. Concomitantly, *L. mucosae* LM410 activated the AhR/STAT3 signaling axis, which likely promoted ISC proliferation and differentiation into goblet cells. These findings demonstrate for the first time that *L. mucosae* may enhance piglet antioxidant defense through coordinated cecal microbiome remodeling, upregulation of tyrosine metabolism and AhR/STAT3-driven epithelial renewal, providing a theoretical foundation for developing *L. mucosae* LM410 as a candidate probiotic to improve piglet health.

## 1. Introduction

Against the background of modern intensive and antibiotic-free farming, the intestinal health of piglets faces severe challenges. Due to their immature digestive and immune systems, weaned piglets are particularly susceptible to oxidative stress. Factors such as weaning, pathogen infection, or heat and cold stress can easily induce oxidative stress, intestinal barrier dysfunction, and dysbiosis in piglets [1,2], which in turn lead to intestinal inflammation, growth retardation, or even death—severely restricting farming efficiency [3]. Current strategies to alleviate oxidative stress and enhance the intestinal barrier in piglets include antibiotics, plant extracts, organic acids, etc. [4]. However, these strategies face limitations such as safety concerns, high costs, and inconsistent efficacy. With the growing emphasis on national policies promoting antibiotic reduction and food safety, novel and sustainable approaches are urgently needed to enhance antioxidant capacity and intestinal barrier function in piglets.

Probiotics, through mechanisms such as improving gut microbiota, enhancing barrier function, and modulating immunity, have emerged as a promising and safe strategy to counteract oxidative stress and improve intestinal health in piglets [5]. Notably, a growing number of studies have been dedicated to investigating the probiotic efficacy of strains derived from indigenous animal breeds. For instance, *Bacillus amyloliquefaciens* strains 40 and TL106, isolated from Jinhua pigs and Tibetan pigs, have been shown to enhance antioxidant and immune function, improve intestinal health and nutrient absorption, and reduce diarrhea in piglets [6,7]. Similarly, Ningxiang pig-derived *Enterococcus hirae* has been shown to exert protective effects against *E. coli*-induced gut dysbiosis and inflammation in piglets [8]. Furthermore, studies have demonstrated that probiotics can remodel the gut microbiota to activate the aryl hydrocarbon receptor/signal transducer and activator of transcription 3 (AhR/STAT3) signaling pathway, driving the proliferation and differentiation of leucine-rich repeat-containing G-protein coupled receptor 5 (Lgr5^+^) intestinal stem cells (ISCs) and thereby facilitating the repair of intestinal epithelial injury in piglets [9,10]. For instance, *Lactiplantibacillus plantarum* has been shown to produce indole-3-lactic acid (ILA) from tryptophan metabolism, which acts as an AhR ligand to activate downstream signaling, including CYP1A1, IL-22, and STAT3, thereby alleviating intestinal inflammation and repairing barrier damage [11]. Similarly, *Lactobacillus rhamnosus GG* metabolites interact with type 3 innate lymphoid cells in the piglet jejunum via AhR, promoting IL-22 secretion and subsequent STAT3 pathway activation, thereby enhancing intestinal barrier function and protecting against pathogen infection [12]. Ren et al. demonstrated in mice that *Lactobacillus paracasei* promotes intestinal mucosal repair by mediating ISCs through gut microbiota-derived metabolites [13]. These findings indicate that *Lactobacillus* may promote intestinal health via AhR/STAT3 pathway activation.

*Limosilactobacillus mucosae* (*L. mucosae*) is a gut-colonizing probiotic known for producing bioactive substances such as antimicrobial peptides, exopolysaccharides, and extracellular enzymes [14,15], which are conducive to enhancing immunity, intestinal barrier integrity, and antioxidant effects [16,17]. Certain strains, including *L. mucosae* LMU1001 and *L. mucosae* AN1, have demonstrated potent antioxidant capacity in model mice and rats through the modulation of antioxidant enzymes (including GSH-PX and T-SOD) and the expression of antioxidant genes (such as *GPX1*, *GPX2*, *MT1*, *MT2*, and *SOD*) and inflammatory cytokine genes (e.g., *IL-6*, *IL-10*, *TNF-α*, *COX-2*, *iNOS*, and *IL-1β*) [18,19]. However, research on the application of *L. mucosae* in livestock is still in its infancy. Only a few studies have indicated that it can improve feed efficiency, intestinal morphology and immune function in commercial pigs [20,21], and one in vitro study showed that *L. mucosae* G01, isolated from Bama pig feces, can protect IPEC-J2 cells against porcine epidemic diarrhea virus [22]. Notably, it remains unclear whether *L. mucosae* can enhance antioxidant capacity and promote the proliferation and differentiation of ISCs in piglets, and the underlying mechanisms have yet to be elucidated.

The Hezuo pig, a subgroup of the plateau-type primitive indigenous Tibetan pig breed, is uniquely adapted to the harsh high-altitude environments and poor feeding and management conditions of China’s pastoral regions [23,24]. Its gastrointestinal microbiota is believed to play a key role in this remarkable adaptability [25]. To screen for potential probiotic candidates, we compared the gut microbiota profiles of Hezuo pigs and Landrace × Yorkshire (LY) pigs, and subsequently isolated, identified, and characterized the strain *L. mucosae* LM410 from Hezuo pig feces. We hypothesized that *L. mucosae* LM410 would improve intestinal barrier function and antioxidant defense through modulation of gut microbiota and activation of the AhR/STAT3 signaling axis. This study aimed to evaluate the effects of different doses of *L. mucosae* LM410 on intestinal morphology, barrier function, antioxidant capacity, and gut microbiota, and to provide preliminary evidence for its potential as a candidate probiotic for improving piglet health.

## 2. Materials and Methods

### 2.1. Pigs and Fecal Sample Collection

For comparison of the intestinal microbiota composition between Hezuo pigs and LY pigs, healthy 6-month-old female Hezuo pigs (*n* = 18; approximate body weight 18–20 kg) and Landrace × Yorkshire (LY) pigs (*n* = 6; approximate body weight 120 kg) were selected. All pigs were confirmed to be free of clinical signs of disease, had no history of antibiotic or probiotic administration for at least 3 months prior to sampling, and had not received any vaccinations during that period. Hezuo pigs were raised under three regimens: semi-grazing in summer (TS, *n* = 6), semi-grazing in winter (TW, *n* = 6), and yard feeding in summer (TS.y, *n* = 6). The inclusion of Hezuo pigs reared under multiple conditions was designed to account for the potential impact of environmental and management practices on gut microbiota. Specifically, we examined whether a uniform differential trend relative to LY pigs emerges under disparate environmental conditions. The semi-grazed Hezuo pigs were reared by farmers in Xiahe County, Gannan Tibetan Autonomous Prefecture, Gansu Province (altitude: approximately 2900 m). They were let out for daily grazing in the morning. In summer, they freely consumed stems, leaves, and seeds of wild plants, including *Potentilla anserina*, *Plantago asiatica*, wild radish and alfalfa, *Poa annua*, and *Cyperaceae.* In winter, they fed mainly on rhizomes of *Potentilla anserina* and other wild plants. Every evening after returning, they were supplemented with a mixed feed consisting of grass meal, corn flour, and highland barley flour. The intensively reared LY pigs (housed in an indoor facility in Lanzhou City, Gansu Province, altitude: approximately 1500 m) and yard-fed Hezuo pigs were sourced from two farms in Lanzhou City, Gansu Province. LY pigs were fed according to the feeding standards for lean-type commercial pigs specified in the National Standard of the People’s Republic of China (GB/T 5915-2020) [26]. Fresh fecal samples were immediately collected into 5 mL sterile centrifuge tubes upon defecation and flash-frozen in liquid nitrogen. These samples were then subjected to 16S rRNA sequencing.

### 2.2. 16S rRNA Gene Sequencing of Fecal Samples

Total bacterial DNA was extracted from fecal samples of Hezuo and LY pigs using the TIANamp Stool DNA Kit (TIANGEN BIOTECH, DP328, Beijing, China). The V3–V4 region of bacterial 16S rRNA genes was amplified with primers 314F (5′-CCTAYGGGRBGCASCAG-3′) and 806R (5′-GGACTACNNGGGTATCTAAAT-3′). Amplicons were purified, pooled, and used to construct sequencing libraries with the TruSeq DNA PCR-Free kits. Libraries were sequenced on the Illumina NovaSeq 6000 platform (2 × 250 bp). Raw reads were demultiplexed and primer-trimmed using QIIME (v1.9.1), assembled using FLASH (v1.2.7), and quality-filtered by truncating at the first base of a continuous low-quality stretch (Phred ≤ 19, length ≥ 3 bp); tags with <75% high-quality bases were discarded. Chimeras were detected and removed using VSEARCH (v2.14.1) against the Silva 132 database. The high-quality reads were clustered into operational taxonomic units (OTUs) at 97% similarity using Uparse (v7.0.1001). Taxonomic annotation was performed using the Mothur pipeline (v3.8.31) with the SSU rRNA database of Silva 132 (http://www.arb-silva.de/, accessed on 5 December 2019). Alpha diversity indices (Observed species, Shannon, Chao1, and ACE) were calculated using QIIME (V1.9.1). Beta diversity was analyzed using Principal Component Analysis (PCA) at the genus level based on Euclidean distance and Non-metric Multi-Dimensional Scaling (NMDS) based on the Bray–Curtis distance, with both performed using R software (v2.15.3). Statistical significance of beta diversity was assessed by permutational multivariate analysis of variance (PERMANOVA) with 999 permutations.

### 2.3. Isolation of L. mucosae LM410 from Hezuo Pigs

Approximately 1 g of fresh fecal samples from Hezuo pigs was suspended in 9 mL of Dulbecco’s Phosphate-Buffered Saline (DPBS; Solarbio, Beijing, China) and serially diluted. Aliquots of 70.00 µL from the 10^−3^, 10^−4^, and 10^−5^ dilutions were spread onto CaCO_3_- MRS agar plates (de Man, Rogosa and Sharpe agar supplemented with 1.0% calcium carbonate and 2.0 mg/mL filter-sterilized vancomycin; Solarbio, Beijing, China). The plates were incubated anaerobically using an anaerobic culture system at 37 °C for 48 h. Colonies exhibiting clear calcium-dissolution rings and typical *Lactobacillus* morphology (milky-white, round, well-defined and convex colonies) were picked and streak-purified three times on MRS agar (Solarbio, Beijing, China) plates. The purified isolates were then subjected to catalase test, Gram staining, microscopic examination, and 16S rRNA gene sequencing. Only isolates that were catalase-negative, Gram-positive, and exhibited typical rod-shaped morphology were retained for further identification. Putative *Lactobacillus* isolates were identified by 16S rRNA gene sequencing using specific primers: LabM-F (5′-TGAGTAACACGTAGGTAACCTG-3′) and LabM-R (5′-ATGCTGATCCGCGATTACT-3′). PCR amplification was performed with the following thermal cycling conditions: initial denaturation at 95 °C for 5 min; followed by 30 cycles of 95 °C for 30 s, 58 °C for 30 s, and 72 °C for 60 s; and a final extension at 72 °C for 10 min. PCR products were electrophoresed on a 1.0% (*w*/*v*) agarose gel. The purified fragments were recovered and submitted to Yangling Tianrun Aoke Biotech Co., Ltd. (Yangling, China) for sequencing. The obtained sequences were aligned with standard strains in the NCBI database using BLAST (v2.16.0) and deposited in GenBank under accession No. MZ047317. A phylogenetic tree was constructed using Mega 6.0 with the Neighbor-Joining method, Kimura 2-parameter model, and 1000 bootstrap replicates, including reference sequences of closely related type strains from the NCBI database. For long-term preservation, overnight cultures of *L. mucosae* LM410 were mixed with sterile glycerol to a final concentration of 30% and stored at −80 °C until further use. 

### 2.4. Whole-Genome Sequencing and ANI Analysis for Species Confirmation

To confirm species identification at the genomic level, whole-genome sequencing of the isolate was performed using a combination of short-read and long-read sequencing technologies. A 400 bp library was constructed and sequenced on the Illumina NovaSeq platform (2 × 150 bp), and a 10 kb library was constructed and sequenced on the PacBio Revio platform. The reads were assembled using Unicycler, Flye, Hifiasm, and Necat, and the assembly was polished with Pilon based on the high-quality short reads. Genome completeness was assessed using CheckM (v1). Average nucleotide identity (ANI) analysis was performed using fastANI against the type strain *Limosilactobacillus mucosae* DSM 13345 (GCF_001436025.1).

### 2.5. Characterization of Probiotic Properties of L. mucosae LM410

Growth curve and acid-production capacity of *L. mucosae* LM410 were determined using the following procedure. The strains were activated and anaerobically cultured in 1.0% MRS broth (Solarbio, Beijing, China) at 37 °C. The OD_600_ and pH values were measured every 2 h to plot growth curves and pH change curves, thereby assessing their acid-production capacity. To evaluate acid tolerance, overnight cultures of *L. mucosae* LM410 were harvested, washed twice with sterile PBS, and resuspended in MRS broth to a final concentration of 1 × 10^8^ CFU/mL. The cell suspensions were incubated for 24 h in MRS broth adjusted to pH 2.0, 3.0, 4.0, and 6.2 (control with uninoculated MRS broth). Bile salt tolerance was assessed by inoculating the same initial concentration of cells (1 × 10^8^ CFU/mL) in MRS broth supplemented with 0%, 0.1%, 0.2%, and 0.3% (*w*/*v*) bile salts (0% served as the control). After 24 h of incubation, viable counts were determined. Survival rates were calculated by comparing the number of viable bacteria after incubation (N) with the initial viable count (N_0_) for each corresponding pH condition, according to the formula:

Survival rate (%) = (N/N_0_) × 100% (1)

The antimicrobial activities of *L. mucosae* LM410 against *Escherichia coli* (ATCC 25922), *Salmonella* (CMCC 50115), *Pasteurella* (ATCC 43137), and *Staphylococcus aureus* (ATCC 25913) were assessed using the Oxford cup agar diffusion method. The cell-free culture supernatant was obtained by centrifuging MRS broth cultures after 24 h anaerobic cultivation, followed by filtration sterilization. The supernatant was used without pH neutralization. As a control, uninoculated MRS broth was adjusted to pH 3.8 using 1 M HCl and tested under the same conditions, following the method described by Letizia et al. [27]. Then, the sterile supernatant was uniformly poured onto agar plates, onto which Oxford cups (8 mm diameter) were placed. Each well was filled with 0.8% Luria–Bertani (LB) agar (Oxoid, Basingstoke, UK) containing 6 lg CFU/mL of indicator bacteria, followed by addition of 100 μL *L. mucosae* LM410 supernatant. Following overnight diffusion at 4 °C and subsequent incubation at 37 °C for 24 h, the inhibitory activity was determined by measuring the diameter of inhibition zones.

The inhibitory effect of *L. mucosae* LM410 on *Clostridium perfringens* type C was further investigated through co-cultivation following previously described methods [28]. The standard strain of *Clostridium perfringens* type C (C59-2, CVCC 2032) was purchased from the China Institute of Veterinary Drug Control (Beijing, China). After overnight cultivation, both strains were centrifuged and washed twice with sterile PBS. The bacterial pellets were resuspended in MRS broth to a final concentration of 2 × 10^7^ CFU/mL. Equal volumes of each suspension were mixed and co-incubated anaerobically at 37 °C for 8 h. To determine the survival of *C. perfringens* type C, aliquots were serially diluted and plated onto LB agar plates, followed by anaerobic incubation. For the enumeration of *L. mucosae* LM410, aliquots were plated onto MRS agar under the same conditions. The selectivity of these media was confirmed by preliminary plating experiments: *L. mucosae* LM410 did not grow on LB agar under the conditions used, whereas *C. perfringens* type C did not grow on MRS agar. Viable counts were determined, and all experiments were repeated three times.

### 2.6. Antioxidant Activity of L. mucosae LM410 In Vitro

After incubation in MRS medium at 37 °C for 24 h, bacterial cells and fermentation supernatant were collected by centrifugation (4 °C, 6000× *g*, 10 min). Fermentation supernatant (FS), intact cell suspension (IC), and cell-free extract (CFE) were prepared following the method of Damayanti et al. [29]. DPPH and ABTS assays were performed using kits (Jiangsu Kete Biotechnology Co., Ltd., Yancheng, China). Samples were mixed with the working solution, and absorbance was measured with a microplate reader (Thermo Fisher Scientific, Waltham, MA, USA) at 517 nm and 734 nm, respectively. The DPPH and ABTS radical scavenging activities were calculated according to the manufacturer’s formulas.

### 2.7. Animal Ethical Statement

The animal experiment protocol was approved by the Animal Care and Use Ethics Committee of Gansu Agricultural University (approval date: 12 March 2024; approval code: GSAU-Eth-AST-2024-024).

### 2.8. Preparation of L. mucosae LM410

The cryopreserved strain was thawed on ice, inoculated (1%, *v*/*v*) into sterile MRS broth (Oxoid, Basingstoke, UK), and incubated anaerobically at 37 °C for 48 h. After incubation, serial dilutions of the culture were spread onto MRS agar plates, which were then incubated anaerobically at 37 °C for 48 h. Colony counts were performed to determine the viable cell count (CFU/mL). Based on the colony counts, bacterial suspensions were adjusted with blank medium to final concentrations of 1 × 10^8^, 1 × 10^9^ and 1 × 10^10^ CFU/mL. Glycerol was added to a final concentration of 20% (*v*/*v*), and the suspensions were dispensed into cryovials and stored at −80 °C until use. Following the method of Chavkin et al. [30], on each day of oral administration, the frozen suspensions were thawed on ice for 1 h and kept on ice throughout the administration process (completed within 1 h after thawing) to maintain bacterial viability.

### 2.9. Piglet Management and Sample Collection

#### 2.9.1. Experimental Design and Animal Management

To investigate the effects of *L. mucosae* LM410 on intestinal health, antioxidant capacity, and microbiota composition in piglets, twelve healthy 28-day-old DLY weaned piglets (half male and half female) with similar body weights (average weight 7.83 ± 0.75 kg) from the same litter were selected from Zhonghui Pig Industry Technology Co., Ltd. (Linxia Prefecture, Gansu Province). Following a 5-day acclimation period, piglets were randomly assigned to four treatment groups (*n* = 3) using a random number table generated in Microsoft Excel, stratified by sex to ensure balanced sex distribution across groups (1–2 males per group). The four groups were fed a basal diet with orally administered *L. mucosae* LM410-free broth (C), or *L. mucosae* LM410 at different doses: low dose (L, 1 × 10^8^ CFU/mL), medium dose (M, 1 × 10^9^ CFU/mL) and high dose (H, 1 × 10^10^ CFU/mL). The piglets were housed in four separate pens (one pen per treatment, with three piglets per pen) under controlled environmental temperature (25 ± 2 °C) at the Animal Training Center, Gansu Agricultural University. Piglets were housed in groups of three per pen, with visual contact between adjacent pens. Each pen was considered the experimental unit for treatment comparisons, while individual piglets served as subsamples for physiological measurements. Prior to the morning feeding each day, piglets were orally administered 3 mL of the corresponding thawed suspension. During the 14-day experimental period, piglets were fed four times daily (08:00, 12:00, 16:00, and 20:00) and had ad libitum access to clean water. The basal diet of piglets was the Anyou Gold Diamond Milk diet (Anyou Biotechnology Group Co., Ltd., Weifang, China), with its ingredients and nutrient composition detailed in Table S1. No antibiotics or vaccinations were administered throughout the study. Throughout the 14 d experimental period, the initial and final body weights of piglets were recorded, and the average daily gain (ADG) was calculated according to the formula: ADG = (final weight − initial weight)/experimental days.

#### 2.9.2. Sample Collection

On the morning of day 15, blood samples were collected from the anterior vena cava of piglets using sterile vacuum tubes. Blood samples were allowed to clot at room temperature for 30 min, centrifuged at 1000× *g* for 10 min at 4 °C, and the serum was aliquoted and stored at −80 °C until analysis of immunological and antioxidant parameters. Piglets were subsequently euthanized by intravenous injection of sodium barbital. Segments of the duodenum, jejunum, and ileum were collected, rinsed with sterile PBS, and fixed in 4% paraformaldehyde for histomorphological analysis. Additional samples of jejunal tissue and cecal content were collected for analysis of gene expression and 16S rRNA sequencing, respectively.

### 2.10. Intestinal Histomorphology Analysis

Segments of the duodenum, jejunum, and ileum fixed with 4% paraformaldehyde were processed through a graded ethanol series, embedded in paraffin, and sectioned. The sections were then stained with hematoxylin and eosin (H&E) and mounted with neutral balsam. Whole-slide images were captured using a Pannoramic 250 digital slide scanner (3DHISTECH, Budapest, Hungary). For each intestinal segment, twenty intact, well-oriented crypt-villus units were selected to minimize selection bias. Villus height (VH) and crypt depth (CD) were measured using Fiji ImageJ 2.9.0 software, and the villus height-to-crypt depth ratio (VCR) was calculated.

### 2.11. Detection of Serum Immunological and Antioxidant Indicators

Concentrations of D-lactic acid (D-LA), complement 3 (C3), and complement 4 (C4) were measured using commercial ELISA kits (Jiangsu Kete Biotechnology Co., Ltd., Jiangsu, China; catalog numbers: D-LA: ADS-W-T017, C3: KT84169-A, C4: KT84172-A), following the manufacturer’s protocols. All kits were validated for use with porcine serum in accordance with the manufacturer’s specifications. Serum total antioxidant capacity (T-AOC), superoxide dismutase (SOD), catalase (CAT), and malondialdehyde (MDA) levels were determined using commercial biochemical assay kits (Jiangsu Kete Biotechnology Co., Ltd., Yancheng, China). Specifically, T-AOC activity was measured using the ferric reducing antioxidant power (FRAP) assay (ADS-W-KY004) and expressed as µmol/mL; SOD activity was measured by the WST-8 method (ADS-W-KY011) and expressed as U/mL; and CAT activity (ADS-W-KY002, expressed as µmol/mL) and MDA level (ADS-W-YH002, expressed as nmol/mL) were assessed using microplate-based assays. All assays were performed in triplicate, and absorbance was measured using a microplate reader (Rayto Life Sciences Co., Ltd., Shenzhen, China).

### 2.12. Real-Time Quantitative PCR (RT-qPCR)

Total RNA was extracted from jejunum tissue using the TRIzol kit (Accurate Biotechnology Co., Ltd., Changsha, China) and reverse-transcribed into cDNA using the Evo M-MLV RT Premix kit (Accurate Biotechnology Co., Ltd., Changsha, China). Primer information used in this study is shown in Table S2. Quantitative real-time PCR (qPCR) was performed using 2 × Universal Blue SYBR Green qPCR Master Mix (Accurate Biotechnology Co., Ltd., Changsha, China) in a 20 µL reaction volume on a QuantGene 9600 (Hangzhou BoRi Technology Co., Ltd., Hangzhou, China). Thermocycling protocols were 95 °C for 30 s, followed by 40 cycles of 95 °C for 5 s and 60 °C for 30 s. Relative gene expression levels were normalized to GAPDH and calculated using the 2^−ΔΔCT^ method.

### 2.13. Immunofluorescence Analysis

Jejunum paraffin sections were dewaxed and rehydrated, followed by fixation, antigen retrieval, and blocking with 1% bovine serum albumin (BSA; A8020, Solarbio, Beijing, China) for 30 min. The sections were then incubated overnight at 4 °C with primary antibodies against Ki67 (41092, Signalway, Greenbelt, MD, USA; 1:200 dilution) and Lgr5 (BS-20746R, Bioss, Beijing, China; 1:200 dilution). After washing, the sections were incubated with the secondary antibody (Alexa Fluor^®^ 488-conjugated goat anti-rabbit IgG; AB150077, Abcam, Cambridge, MA, USA; 1:1000 dilution). For negative control staining, representative sections were processed in parallel with the primary antibody omitted and replaced by PBS to confirm the specificity of secondary antibody binding. Finally, nuclei were counterstained with DAPI (BL105B, Biosharp, Beijing, China), and the sections were mounted using an anti-fluorescence quenching mounting medium (BL701A, Biosharp, Beijing, China). Images were acquired using a panoramic slide scanner (Pannoramic MIDI II, 3DHISTECH, Budapest, Hungary).

For quantitative analysis, three random fields of view (×200 magnification) per section were selected using panoramic viewing software for photography, while areas with poor tissue integrity were avoided. The acquired images were analyzed with Image Pro Plus software (v6.0) to measure the integrated fluorescence intensity and the area of protein expression, from which the mean fluorescence intensity was calculated. Statistical analysis and graphing were performed using GraphPad Prism software.

### 2.14. Western Blot Analysis

Jejunum tissue proteins were extracted using high-strength RIPA lysis buffer (Servicebio, Wuhan, China; G2002-100ML) supplemented with phosphatase and protease inhibitors (Servicebio, Wuhan, China; G2007-1ML). Total protein concentration was determined with a BCA protein assay kit (Servicebio, Wuhan, China; G2026-200T). Protein samples (30 µg per lane) were mixed with 5× protein loading buffer (Servicebio, Wuhan, China; G2075-100ML), denatured at 95 °C for 10 min, separated by 10% sodium dodecyl sulfate-polyacrylamide gel electrophoresis (SDS-PAGE; Servicebio, Wuhan, China; G2043-50T), and transferred onto polyvinylidene difluoride (PVDF) membranes (Servicebio, Wuhan, China; WGPVDF45). Membranes were blocked with rapid blocking buffer at room temperature for 10 min, then incubated overnight at 4 °C with primary antibodies against AhR (Servicebio, Wuhan, China; GB115154; 1:800 dilution), p-STAT3 (Servicebio, Wuhan, China; GB150001; 1:1000 dilution), STAT3 (Servicebio, Wuhan, China; GB150216; 1:1000 dilution), and GAPDH (Servicebio, Wuhan, China; GB15003; 1:5000 dilution), diluted in TBST (Servicebio, Wuhan, China; G2150-1L) at appropriate ratios. Following three washes with TBST, membranes were incubated with horseradish peroxidase (HRP)-conjugated goat anti-rabbit IgG secondary antibody (Servicebio, Wuhan, China; GB23303; 1:3000 dilution) for 30 min at room temperature. Immunoreactive bands were visualized using an enhanced chemiluminescence (ECL) kit (Servicebio, Wuhan, China; G2020-25ML), applied to membranes for 1 min prior to imaging using a chemiluminescence imaging system (SCG-W3000, Servicebio, Wuhan, China). Images were quantitatively analyzed with AIWBwell^TM^ software (v8.0.2.2, Servicebio, Wuhan, China), and protein expression levels were normalized to GAPDH. All Western blot analyses were performed with three independent biological replicates (*n* = 3 per group).

### 2.15. 16S rRNA Gene Sequencing of Cecal Contents

Total bacterial DNA was extracted from cecal content samples of piglets using the MagBeads FastDNA Kit (MP Biomedicals, Navi Mumbai, India). DNA purity and concentration were assessed by NanoDrop spectrophotometry, agarose gel electrophoresis, and Qubit fluorometry. Amplification was performed with primers 338F (5′-barcode + ACTCCTACGGGAGGCAGCA-3′) and 806R (5′-GGACTACHVGGGTWTCTAAT-3′). Amplicons were purified, pooled, and used to construct sequencing libraries with the TruSeq DNA PCR-Free and TruSeq Nano DNA LT kits. Libraries were sequenced on the Illumina NovaSeq 6000 platform (2 × 250 bp). Raw reads were processed using QIIME2 (version 2024.5) following the official tutorial (https://docs.qiime2.org/, accessed on 3 April 2025). Raw sequence data were demultiplexed using the q2-demux plugin, and primer sequences were trimmed using the q2-cutadapt plugin. Denoising, quality filtering, read merging, and chimera removal were performed using the q2-dada2 plugin. Clean reads were merged into amplicon sequence variants (ASVs) at 100% sequence similarity, and an abundance table was generated. Taxonomy was assigned by aligning ASV representative sequences against the Greengenes2 database. Beta diversity was analyzed by principal coordinates analysis (PCoA) based on weighted UniFrac distance and statistically tested using permutational multivariate analysis of variance (PERMANOVA) with 999 permutations. Alpha diversity indices (Chao1, Shannon, Simpson, Observed species) were computed using QIIME2; rarefaction curves were generated, and inter-group differences at the phylum and genus levels were assessed by linear discriminant analysis effect size (LEfSe). Bar plots at each taxonomic level were generated using R software (v4.3.3). Functional predictions were inferred using Phylogenetic Investigation of Communities by Reconstruction of Unobserved States 2 (PICRUSt2, v2.2.2-b) based on the Kyoto Encyclopedia of Genes and Genomes (KEGG) database (14 significantly different pathways visualized), and Spearman correlation heatmaps were generated using OmicStudio (https://www.omicstudio.cn).

### 2.16. Statistical Analysis

Intestinal morphology measurements, barrier function data, immune parameters, and antioxidant enzyme activity were analyzed using one-way analysis of variance (ANOVA), followed by Fisher’s Least Significant Difference (LSD) post hoc tests for multiple comparisons using SPSS software (v27.0). For the analysis of strain characteristics and microbial comparisons among the four treatment groups, one-way ANOVA followed by LSD post hoc test was performed. For comparisons between two groups, an independent-samples *t*-test was used. Results are presented as mean ± standard deviation (SD). Graphs were generated using GraphPad Prism (v9.0). A *p*-value below 0.05 was considered statistically significant.

For 16S rRNA sequencing data, Alpha diversity indices were compared using Student’s *t*-test for two-group comparisons or one-way ANOVA followed by the LSD post hoc test for four-group comparisons. Principal Component Analysis (PCA) based on Euclidean distance and Non-metric Multi-dimensional Scaling (NMDS) using R software (v3.6.3) based on Bray–Curtis distance were applied to assess the similarity of microbial community structures across different samples. Differential microbial taxa were identified using LEfSe, with a logarithmic LDA score threshold of >2.0. Functional predictions were analyzed using PICRUSt2, with significantly different KEGG pathways identified by Student’s *t*-test. Spearman’s rank correlation analyses were performed using the SparCC (Sparse Correlations for Compositional Data) method implemented in the sparcc package within R software (v3.6.3).

## 3. Results

### 3.1. Hezuo Pigs Exhibited Greater Bacterial Diversity and Higher Relative Abundance of L. mucosae than LY Pigs

**Figure 1:**
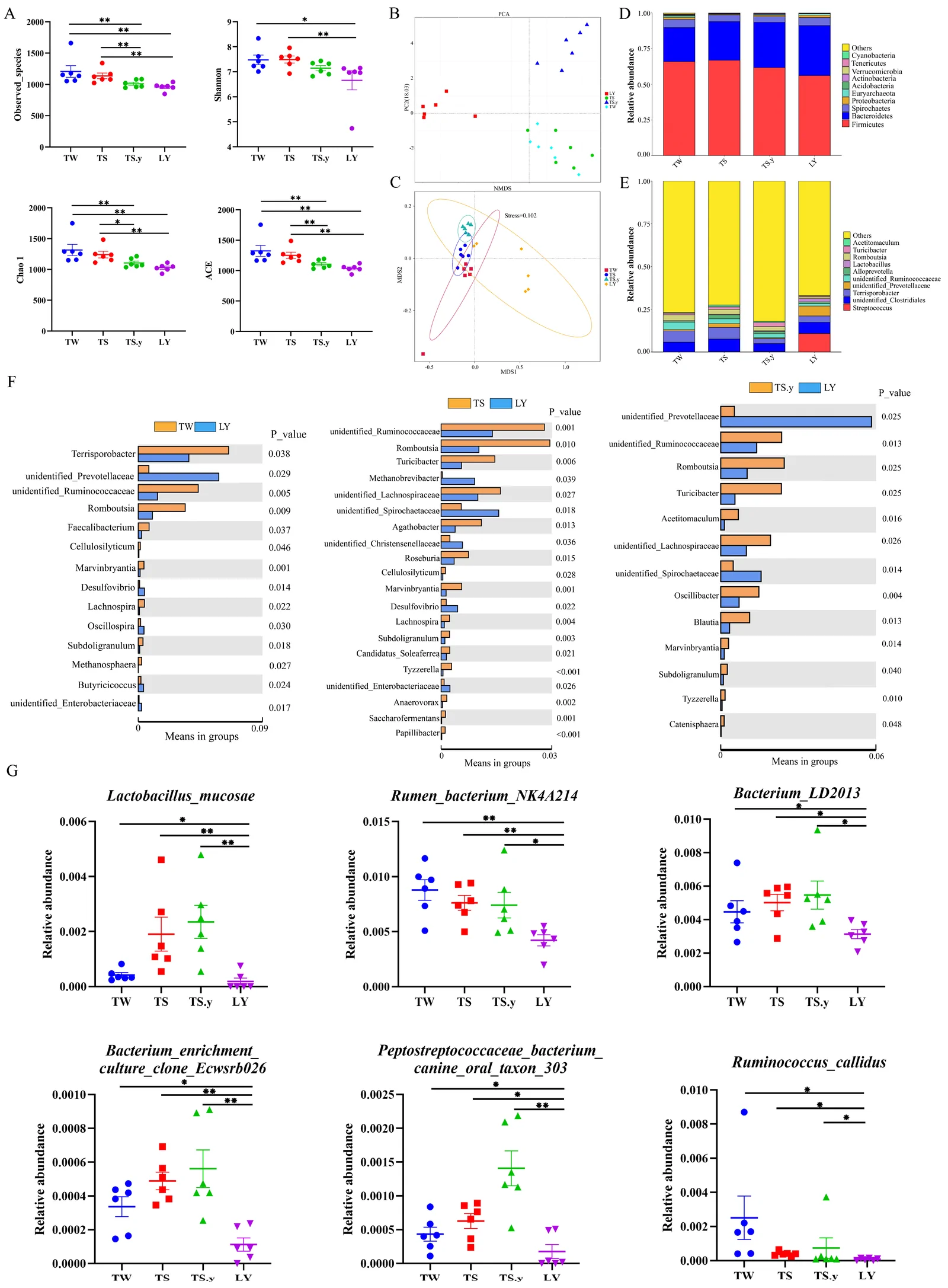
Gut microbiota diversity and composition between Hezuo and LY pigs. (**A**) Alpha diversity indices (Observed_species, Shannon, Chao1, and ACE). (**B**) PCA based on Euclidean distances at the genus level; (**C**) NMDS based on Bray–Curtis distance at the OTU level. (**D**,**E**) Relative abundance of the top 10 microbial taxa at the phylum and genus levels, respectively. (**F**) Significantly different genera between groups. (**G**) Key bacterial species exhibiting significant differences among groups. (Data are presented as the mean ± standard deviation (SD) (*n* = 6). * *p* < 0.05, ** *p* < 0.01; One-way ANOVA for (**A**,**G**), *t*-test for (**F**)).

Comparative 16S rRNA sequencing analysis of fecal microbiota revealed that Hezuo pigs exhibited significantly richer microbial diversity than LY pigs, as indicated by higher Observed_species, Shannon, Chao1, and ACE indices in semi-grazing Hezuo pigs during both summer and winter (TS and TW groups) (Figure 1A). The results of PCA and NMDS revealed a clear distinction in the microbiota between LY pigs and Hezuo pig (TS, TS.y, and TW groups) (Figure 1B,C). At the phylum level, the microbiota of both Hezuo and LY pigs were primarily composed of Firmicutes and Bacteroidete. Compared to LY pigs, Hezuo pigs exhibited a higher abundance of Firmicutes and a lower relative abundance of Proteobacteria (Figure 1D). At the genus level, both Hezuo and LY pigs showed a predominant abundance of unidentified_Clostridiales and Terrisporobacter. Notably, Hezuo pigs were significantly enriched in unidentified_Ruminococcaceae, Alloprevotella, and Romboutsia, which are known for inhibiting pathogens and producing short-chain fatty acids (SCFAs). In contrast, LY pigs exhibited higher relative abundances of Streptococcus and unidentified_Prevotellaceae (Figure 1E). The significant differences among groups at the genus level are shown in Figure 1F. Compared to LY pigs, Hezuo pigs (TW, TS, TS.y groups) displayed significantly higher relative abundances of unidentified_Ruminococcaceae, Romboutsia, Marvinbryantia, and Subdoligranulum. Genera such as Turicibacter, unidentified_Lachnospiraceae, and Tyzzerella were also more abundant in the TS and TS.y Hezuo pigs; unidentified_Ruminococcaceae, Romboutsia, Cellulosilyticum, Marvinbryantia, Lachnospira, and Subdoligranulum were also more abundant in the TW and TS Hezuo pigs. Conversely, the LY pigs exhibited significantly higher abundances of unidentified_Spirochaetaceae, unidentified_Prevotellaceae, Desulfovibrio, and unidentified_Enterobacteriaceae compared to Hezuo pigs. Species-level analysis revealed that *Lactobacillus mucosae*, *Rumen bacterium* NK4A214, *Bacterium* LD2013, *Bacterium enrichment culture clone* Ecwsrb026, *Peptostreptococcaceae bacterium canine oral* taxon303 and *Ruminococcus_callidus* were present at significantly higher relative abundances in the fecal microbiota of the Hezuo pig (TW, TS, TS.y) groups compared with LY pigs (Figure 1G). Among these, *L. mucosae* is particularly noteworthy for its reported roles in maintaining intestinal barrier integrity and antagonizing pathogenic bacteria [31,32,33]. Therefore, the higher abundance of *L. mucosae* in Hezuo pigs motivated us to further isolate and characterize this strain to assess its probiotic potential.

### 3.2. Isolation, Identification, and Biological Characteristics of L. mucosae LM410

On MRS agar, Lactobacillus isolates exhibited characteristic colony morphology: milky-white, round with smooth margins, and convex (Figure S1A). Following exposure to hydrogen peroxide, catalase-negative isolates lacking bubble formation were excluded (Figure S1B). Strains conforming to the morphological features of Gram-positive, rod-shaped, non-spore-forming, and non-motile bacteria were identified via Gram staining and oil-immersion microscopy (Figure S1C). HBI rapid identification strips further narrowed the isolates to those with biochemical profiles consistent with *Lactobacillus* (Table S3).

**Figure 2:**
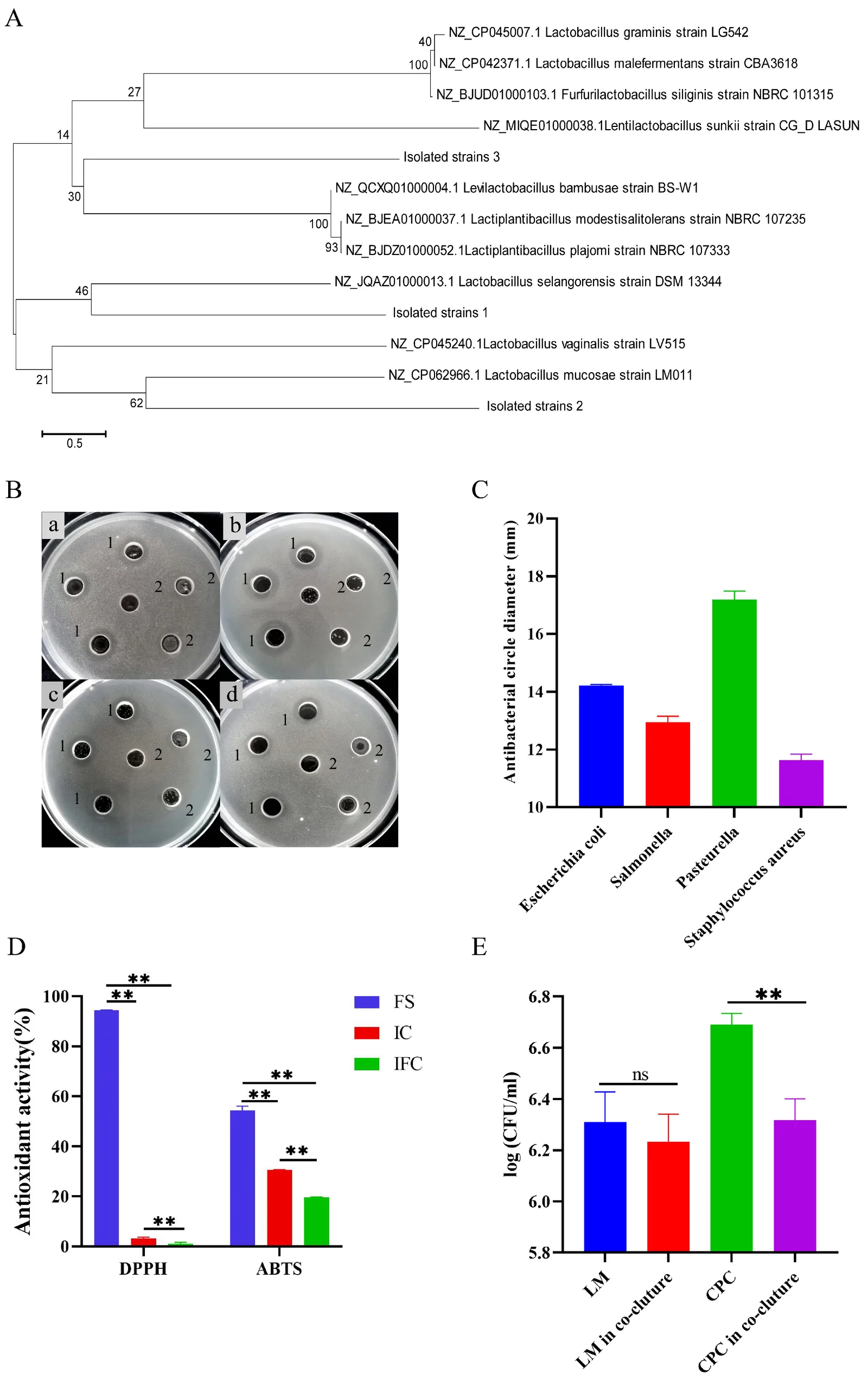
Phylogenetic tree, inhibitory and antioxidant activities of *L. mucosae* LM410. (**A**) Phylogenetic tree based on 16S rRNA gene sequences of the candidate strains. (**B**) Antagonistic activity of *L. mucosae* LM410 against common pathogenic bacteria. 1: *L. mucosae* LM410 fermentation broth; 2: Blank control; (**a**): *Escherichia coli*; (**b**): *Pasteurella*; (**c**): *Salmonella*; (**d**): *Staphylococcus aureus*. (**C**) Antibacterial circle diameter. (**D**) Antioxidant activity of *L. mucosae* LM410 in vitro. FS: fermented supernatant, IC: intact cells, CFE: cell-free extracts. (**E**) *L. mucosae* LM410 inhibition capacity against *CpC* (LM: LM410 viable cell count; LM in co-culture: LM410 viable cell count in LM410-*CpC* co-culture; CPC: *CpC* viable cell count; *CPC* in co-culture: *CpC* viable cell count in LM410-*CpC* co-culture). ** *p* < 0.01, ns indicates not significant; One-way ANOVA for (**C**–**E**).

PCR targeting the Lactobacillus-specific 16S rRNA gene yielded a single clear band of approximately 1261 bp for three of the isolates on agarose gel (Figure S1D). Sequencing of the amplicons and phylogenetic tree analysis revealed that isolated strain 2 exhibited the highest homology to *Lactobacillus mucosae* LM011 (NZ_CP062966.1), with 100% coverage and 99% sequence identity (Figure 2A). To further confirm the species identification at the genome level, whole-genome sequencing of isolate 2 was performed. The genome was assembled into a complete circular chromosome of 2,075,737 bp with a GC content of 46.80% and one plasmid of 69,082 bp (43.32% GC), with a CheckM completeness of 99.18%. ANI analysis against the type strain *Limosilactobacillus mucosae* DSM 13345 (GCF_001436025.1) yielded an ANI value of 96.07%, exceeding the ≥95% species delineation threshold. Based on integrated analyses including whole-genome sequencing and ANI, isolated strain 2 was identified and designated as *Lactobacillus mucosae* LM410. Of note, the species *Lactobacillus mucosae* was reclassified as *Limosilactobacillus mucosae* in 2020 [34]. Its 16S rRNA sequence has been deposited in the NCBI database (accession NO. MZ047317), and the strain is preserved in the China General Microbiological Culture Collection Center (CGMCC No. 22828). The complete genome sequence of *L. mucosae* LM410 has been deposited in the NCBI GenBank database under accession number PRJNA1414803.

The growth curve of *L. mucosae* LM410 showed that the strain was in the lag phase from 0 h to 8 h. After 8 h, it entered the rapid exponential phase from 8 h onward, reaching a peak optical density value of 4.16 at 20 h of culture, followed by the stationary phase and then gradual decline (Figure S1E). Acid production of *L. mucosae* LM410 was robust, causing a sharp pH decline during the first 2 h of cultivation and reaching below 4.50 by 22 h, after which it stabilized (Figure S1F). The strain also demonstrated strong environmental stress tolerance, retaining viability rates exceeding 80% in MRS broth at pH 2.00–4.00 (Table S4) and over 93% survival in MRS broth supplemented with 0.10–0.30% bile salts (Table S5). *L. mucosae* LM410 showed antagonistic activity against four common pathogenic bacteria: *Escherichia coli*, *Pasteurella*, *Salmonella* and *Staphylococcus aureus*, producing clear inhibition zones on agar plates with mean diameters exceeding 10.00 mm (Figure 2B,C). Notably, its strongest inhibitory effect was observed against *Pasteurella*. The pH-adjusted MRS control (pH 3.8) did not produce any inhibition zone, indicating that the observed antimicrobial activity was not solely attributable to organic acid production. In vitro antioxidant activity assays of *L. mucosae* LM410 cultures showed that the FS group had the highest DPPH radical scavenging activity (94.38%), significantly exceeding the IC (3.14%) and CFE (1.09%) groups (*p* < 0.01). LM410 also exhibited considerable ABTS radical scavenging capacity, with the FS group again significantly outperforming the IC and CFE groups (*p* < 0.01), reaching a scavenging rate of 54.42% (Figure 2D). Co-culture of *L. mucosae* LM410 with *Clostridium perfringens* type C (*CpC*) resulted in a significant reduction in the viable count of *CpC*, further confirming its inhibitory effect against pathogenic bacteria (Figure 2E).

### 3.3. L. mucosae LM410 Supplementation Suggested Improved Intestinal Morphology and Barrier Function Compared with the Control Group of DLY Weaned Piglets

**Figure 3:**
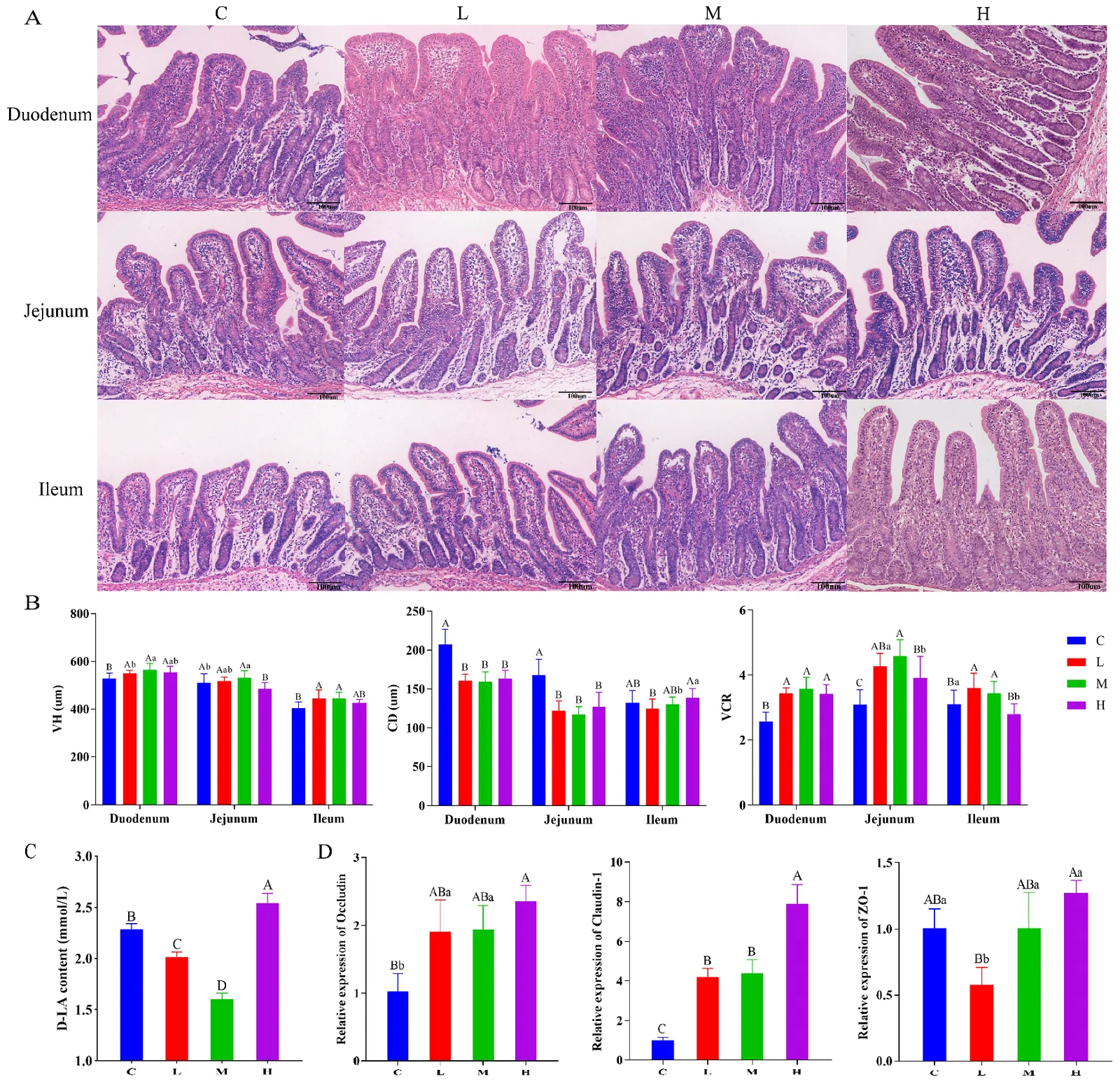
*L. mucosae* LM410 supplementation suggested improved intestinal morphology and barrier function of DLY piglets. (**A**) H&E staining of duodenum, jejunum and ileum (150×), scale bar: 100 µm. (**B**) Villus height, crypt depth and villus height/crypt depth ratio. (**C**) Serum D-lactic acid content. (**D**) The relative expression of the tight junction proteins *Occludin*, *Claudin-1*, and *ZO-1* in the jejunum. Data are presented as the mean ± standard deviation (SD) (*n* = 3). Different lowercase letters indicate significant difference (*p* < 0.05); different uppercase letters indicate highly significant difference (*p* < 0.01).

Based on prior characterization, we conducted a 14-day animal trial to further investigate the effects of different concentrations of *L. mucosae* LM410 on the weight gain, intestinal morphology, and barrier function of DLY weaned piglets. *L. mucosae* LM410 supplementation did not significantly alter overall growth performance (*p* > 0.05, Table S6). Histological analysis of the duodenum, jejunum, and ileum of piglets showed that low and medium doses of *L. mucosae* LM410 improved the intestinal villus structure and mucosal integrity, whereas the high dose of *L. mucosae* LM410 caused obvious intestinal injury and villus fragmentation (Figure 3A,B). Compared with the control group, the VH was significantly increased in the duodenum (all doses, *p* < 0.01), jejunum (medium dose, *p* < 0.05) and ileum (low and medium doses, *p* < 0.01) in groups supplemented with *L. mucosae* LM410, but decreased in the high-dose group of the jejunum (*p* < 0.01). The CDs were significantly decreased, and the VCRs were significantly elevated in the duodenum and jejunum of all *L. mucosae* LM410-supplemented groups (*p* < 0.01). Of note, the CD was significantly increased while the VCR was significantly decreased in the ileum of group H (*p* < 0.05).

*L. mucosae* LM410 also enhanced gut barrier function. Serum D-LA is a marker of intestinal permeability. Compared with the C group, serum D-LA levels were significantly decreased in the L and M groups (*p* < 0.01; Figure 3C), with the most pronounced effect observed in the M group (*p* < 0.01). In contrast, this indicator was significantly increased in the H group (*p* < 0.01). Furthermore, in all *L. mucosae* LM410 treatment groups, the mRNA expression levels of key tight junction proteins *Occludin* and *Claudin-1* were significantly upregulated; only the H group showed an upregulation of *ZO-1* expression (*p* < 0.05; Figure 3D). Collectively, these findings suggest that low and medium doses of *L. mucosae* LM410 can profoundly improve intestinal morphology and barrier function in weaned piglets, whereas a high dose may exert detrimental effects. Notably, a clear dose-dependent response was observed: low and medium doses of *L. mucosae* LM410 exerted beneficial intestinal effects, whereas the highest dose resulted in adverse outcomes. This finding highlights the dose sensitivity of *L. mucosae* LM410 and provides crucial evidence for establishing a safe and effective dosage range for its application in weaned piglets.

### 3.4. Medium-Dose L. mucosae LM410 Was Associated with Enhanced Immune and Antioxidant Capacity of DLY Weaned Piglets

**Figure 4:**
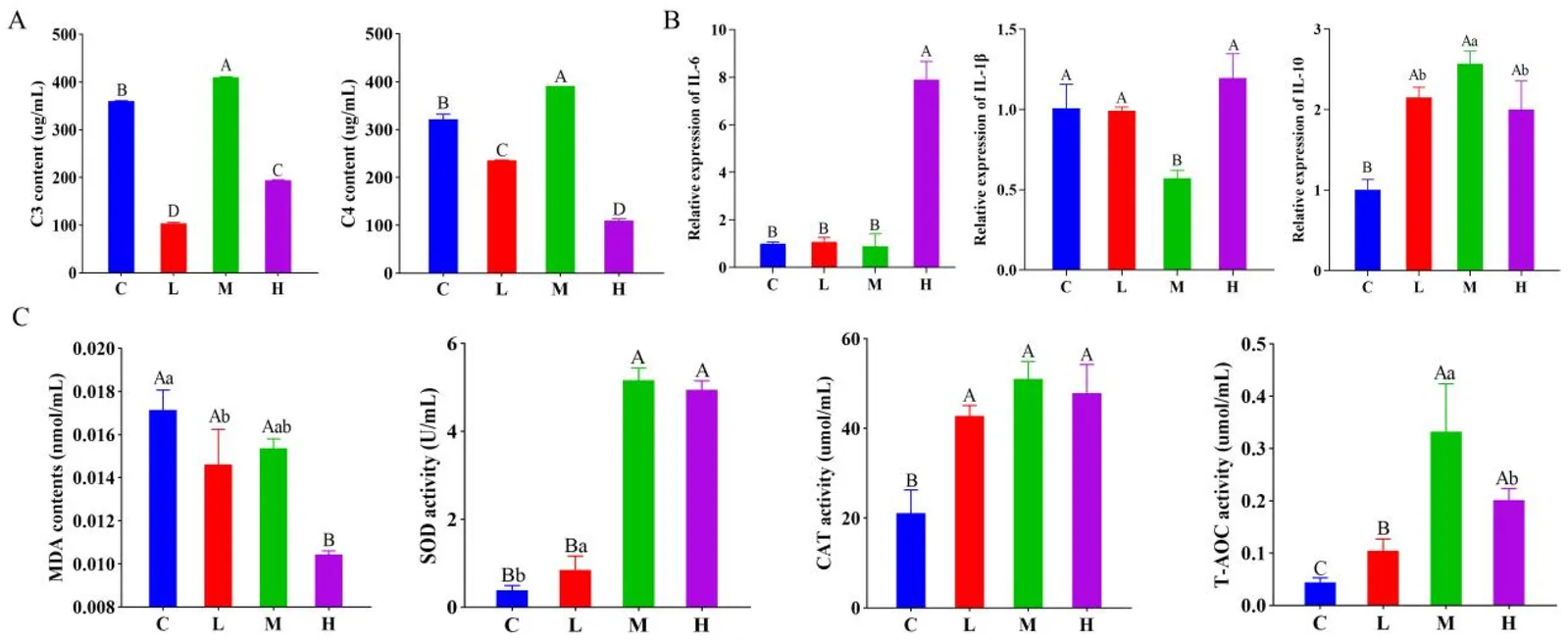
*L. mucosae* LM410 was associated with enhanced immunity and antioxidant capacity in DLY piglets. (**A**) Serum C3 and C4 content. (**B**) The relative expression of inflammatory factors *IL-6*, *IL-1β*, and *IL-10* in jejunal tissue. (**C**) Serum antioxidant enzyme activity. Data are presented as the mean ± standard deviation (SD) (*n* = 3). Different lowercase letters indicate significant difference (*p* < 0.05); different uppercase letters indicate highly significant difference (*p* < 0.01).

Serum complement and cytokine analyses revealed a bell-shaped dose–response effect of *L. mucosae* LM410 on the immune and antioxidant capacity of weaned piglets (Figure 4A,B). Compared with the C group, C3 and C4 contents were significantly increased only in the M group, while significantly decreased in the L and H groups (*p* < 0.01; Figure 4A). Regarding inflammatory cytokines, the expression of pro-inflammatory cytokines interleukin-6 (*IL-6)* and interleukin-1β (*IL-1β*) was significantly increased in the H group (*p* < 0.01). In contrast, *IL-1β* expression was significantly decreased in the M group (*p* < 0.01), whereas the anti-inflammatory interleukin-10 (*IL-10*) was significantly increased in all LM410 treatment groups (*p* < 0.01), with the highest expression level observed in the M group (Figure 4B). Supplementation with *L. mucosae* LM410 also enhanced systemic antioxidant capacity in piglets (Figure 4C). Serum MDA content was significantly decreased in the L and H groups (*p* < 0.05). The activities of antioxidant enzymes SOD and CAT, as well as total antioxidant capacity T-AOC, were significantly enhanced in all *L. mucosae* LM410-treated groups, with the highest values of these parameters observed in the M group. Collectively, these results indicate that the beneficial immunomodulatory effects of *L. mucosae* LM410 were primarily observed at the medium dose, whereas the high dose induced a potentially pro-inflammatory response. This dose-dependent pattern underscores the importance of dose optimization when considering the immunomodulatory application of *L. mucosae* LM410 in piglets.

### 3.5. Medium-Dose L. mucosae LM410 Modulated Cecal Microbiota Composition and Enriched SCFA-Producing Bacteria in DLY Weaned Piglets

Taken together, the above findings revealed that medium-dose *L. mucosae* LM410 was associated with the most prominent effects on improving piglet health. Based on this, cecal contents from the C group and M group were subjected to 16S rRNA sequencing to investigate the effect of *L. mucosae* LM410 on the gut microbiota of piglets. Sequencing generated 583,626 raw reads, yielding 368,867 high-quality sequences after quality processing, with sufficient sequencing coverage confirmed for reliable analysis by species accumulation curves (Figure S2). A total of 21 phyla, 30 classes, 65 orders, 124 families, 375 genera and 599 species were annotated.

**Figure 5:**
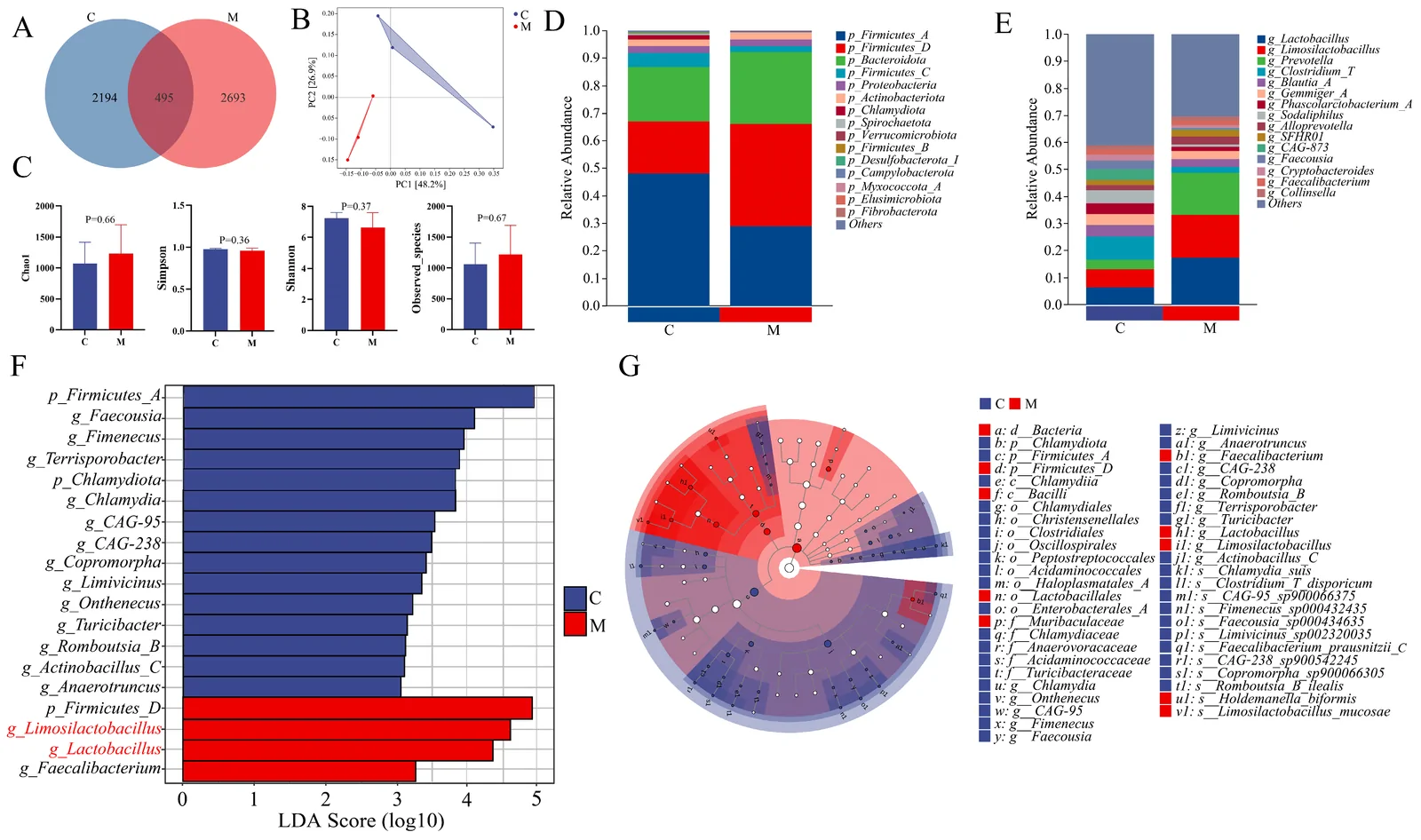
Effect of *L. mucosae* LM410 on the gut microbiota of DLY piglets (*n* = 3). (**A**) Venn diagram of unique ASVs. (**B**) PCoA based on weighted UniFrac distances. (**C**) Alpha diversity analysis plot. Significance analysis was conducted using Student’s *t*-test. (**D**,**E**) Relative abundance of the top 15 microbial phyla and the top 15 genera. (**F**) Histogram of LEfSe analysis at phylum and genus levels. (**G**) Cladogram from phylum to genus. (Kruskal–Wallis test *p* < 0.05 and LDA score (log10) > 3.0). *n* = 3.

Although α-diversity indices demonstrated no significant intergroup differences, *L. mucosae* LM410 administration exhibited a higher number of unique ASVs than the control group (2693 vs. 2194, Figure 5A,C). Beta-diversity based on phylogenetic relationships between the two groups was evaluated using PCoA with Weighted UniFrac distances (Figure 5B). The results showed that the gut microbial communities of the C and M groups were clearly separated along the first principal coordinate (PC1), indicating a distinct niche differentiation between them. Furthermore, PCoA based on Bray–Curtis distances also confirmed the group differences in terms of species composition and abundance (Figure S3A). At the phylum level (Figure 5D), Firmicutes (including Firmicutes_*A*, *B*, *C*, and *D*) and *Bacteroidetes* accounted for over 90% of the microbiota in both groups. Supplementation with *L. mucosae* LM410 increased the relative abundances of Firmicutes_D and Bacteroidetes in the cecum of weaned piglets, while the abundance of Chlamydiota was significantly higher than that in the C group, where it was almost undetectable (approximately 0%). At the genus level (Figure 5E), Prevotella, Lactobacillus and Limosilactobacillus were the three dominant genera in group M. The combined abundance of Lactobacillus and Limosilactobacillus was markedly higher in group M (33.1%) than in group C (12.9%), whereas Clostridium_T was the dominant genus in group C (8.4%).

LEfSe analysis identified a total of 48 differential microbial biomarkers between the two groups (Figure 5G). The taxa at the phylum and genus levels are presented in the LEfSe plot (Figure 5F). Notably, only 3 genera Limosilactobacillus, Lactobacillus, and Faecalibacterium were enriched in group M, whereas 13 genera were enriched in group C, including Faecousia, Fimenecus, Terrisporobacter, Chlamydia, CAG-95, CAG-238, Limivicinus, Onthenecus, Actinobacillus, Copromorpha, Romboutsia_B, Turicibacter, and Anaerotruncus. This suggests that *L. mucosae* LM410 may enhance its competitive advantage within the microbiota, reshape the gut microbial community structure, and shape the microbiota toward a more beneficial composition. Consistently, the differential analysis at the species level further supported this finding (Figure S3B). The relative abundances of *L. mucosae* and *Holdemanella biformis* were significantly increased in group M (*p* < 0.05), while several potentially harmful species, such as *Clostridium_T disporicum* and *Chlamydia suis*, were significantly decreased compared with the control group (*p* < 0.05).

### 3.6. L. mucosae LM410 Supplementation Was Associated with Intestinal Barrier and Antioxidant Function and Upregulated Tyrosine Metabolism

**Figure 6:**
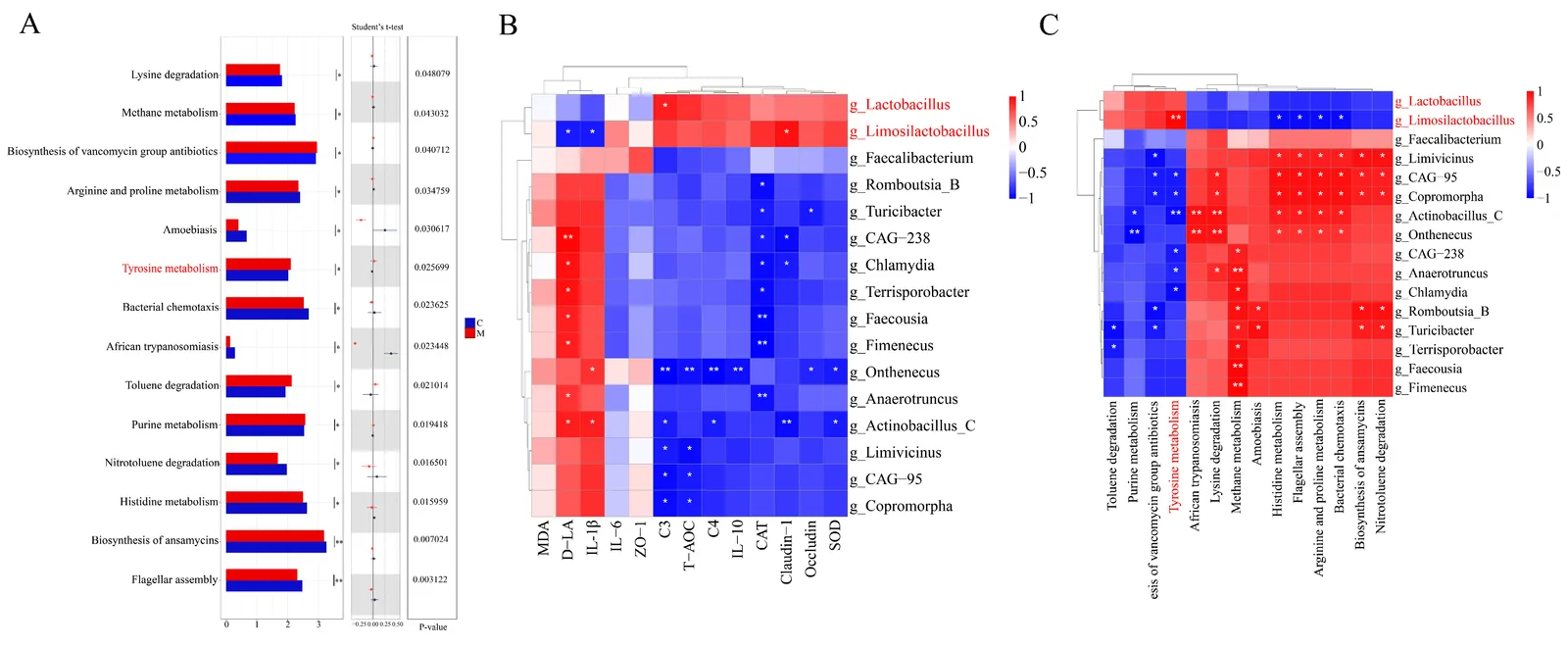
*L. mucosae* LM410 supplementation was associated with intestinal barrier and antioxidant function and upregulated tyrosine metabolism. (**A**) Differential functions of the cecal microbiota from piglets between group C and group M using PICRUSt2 prediction. Student’s *t*-test was performed for significance analysis. (**B**) Correlation analysis between differential bacterial genera and biochemical indicators. (**C**) Correlation analysis between differential bacterial genera and KEGG metabolic pathways. *n* = 3. * *p* < 0.05, ** *p* < 0.01.

To further explore the functions of microbial biomarkers, we performed PICRUSt2 functional prediction on differential microbiota and analyzed their correlations with intestinal barrier and antioxidant indices. Compared with group C, purine metabolism, tyrosine metabolism, and toluene degradation were significantly upregulated in group M, whereas pathways associated with bacterial motility (flagellar assembly and bacterial chemotaxis) and specific amino acid metabolisms pathways (histidine metabolism and arginine metabolism) were downregulated (*p* < 0.05, Figure 6A). Correlation analysis revealed that the key genera *Lactobacillus* and *Limosilactobacillus* showed strong positive correlations with C3 and *Claudin-1* (*p* < 0.05), and were negatively correlated with the intestinal permeability marker D-LA and the pro-inflammatory cytokine *IL-1β* (*p* < 0.05). In contrast, other bacterial genera with significantly reduced abundance in group M were positively correlated with intestinal inflammation and permeability markers (D-LA, *IL-1β*) and negatively correlated with antioxidant enzymes (SOD, T-AOC), tight junction proteins (*Occludin*, *Claudin-1*), and immune factors (*IL-10*, C3, C4) (Figure 6B). This suggests that the enrichment of Lactobacillus and Limosilactobacillus may contribute to enhancing intestinal epithelial barrier function and systemic antioxidant activity of piglets by competitively inhibiting the proliferation of other potentially harmful bacteria (such as *Onthenecus*). Correlation analysis between differential microbiota and their functions revealed that Limosilactobacillus was significantly positively correlated with the tyrosine metabolism pathway (*p* < 0.01), while Limivicinus, CAG-95, Copromorpha, Actinobacterium C and Onthenecus were negatively correlated with the tyrosine metabolism pathway (*p* < 0.05; Figure 6C). These findings suggest that *L. mucosae* LM410 may reinforce systemic antioxidant capacity and intestinal barrier function and was associated with reshaping of the intestinal microbiota and upregulation of tyrosine metabolism in piglets.

### 3.7. L. mucosae LM410 Supplementation Was Associated with ISC Proliferation and Differentiation and Activation of the AhR/STAT3 Signaling Pathway

**Figure 7:**
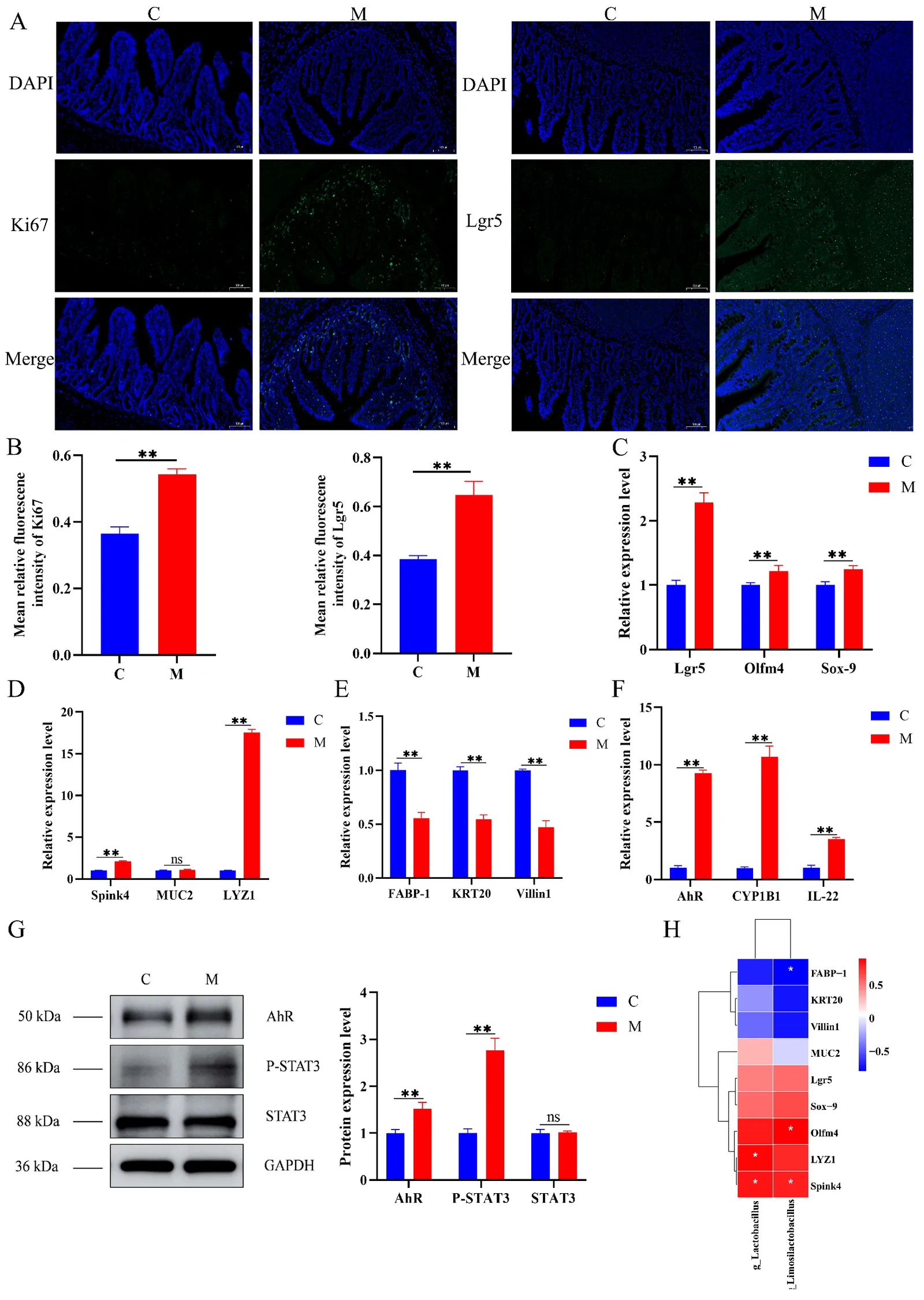
*L. mucosae* LM410 supplementation was associated with ISC proliferation and differentiation and activation of the AhR/STAT3 signaling pathway. (**A**) Representative immunofluorescence staining images of Ki67 and Lgr5 in jejunal tissue. (**B**) The relative fluorescence intensity of Ki67 and Lgr5. (**C**) Relative mRNA expression of intestinal stem cell marker genes (*Lgr5*, *Olfm4, Sox-9*). (**D**) Relative mRNA expression of goblet cell marker genes (*Spink4*, *MUC2*) and the Pan-cell marker gene (*LYZ1*). (**E**) Relative mRNA expression of the enterocyte cell marker genes (*FABP-1*, *KRT20*) and the absorptive enterocyte cell marker gene (*Villin1*). (**F**) Relative mRNA expression of *AhR*, *CYP1B1* and *IL-22*. (**G**) Protein expression of AhR, p-STAT3 and STAT3. (**H**) Correlation analysis between differential bacterial genera and gut stem cell marker genes. Data are presented as the mean ± standard deviation (SD) (*n* = 3). ** *p* < 0.01, ns indicates not significant.

Immunofluorescence staining of the ISC marker Lgr5 revealed that oral administration of *L. mucosae* LM410 significantly promoted ISC proliferation in piglets (*p* < 0.05, Figure 7A,B) and upregulated the mRNA expression levels of ISC marker genes *Lgr5*, *Olfm4*, and *Sox-9* (*p* < 0.05), as well as goblet cell and Paneth cell marker genes *LYZ1* and *Spink4* (*p* < 0.01) (Figure 7C,D). Meanwhile, the mRNA expression of enterocyte marker genes (*FABP1* and *KRT20*), as well as the absorptive enterocyte marker gene *Villin1*, was significantly downregulated (*p* < 0.01, Figure 7E). These results indicate that *L. mucosae* LM410 was associated with the proliferation and expansion of ISCs, differentiation into goblet cells, and reduced differentiation into absorptive enterocytes.

To investigate whether *L. mucosae* LM410 was associated with activation of the intestinal AhR/STAT3 signaling pathway in the jejunum of piglets, RT-qPCR and Western blot analyses were performed. RT-qPCR results showed that oral administration of *L. mucosae* LM410 significantly upregulated the mRNA expression of key genes *AhR*, *CYP1A1*, and *IL-22* in this pathway (*p* < 0.01, Figure 7F). Furthermore, Western blot revealed that the protein expression levels of AhR and p-STAT3 were also significantly increased (*p* < 0.01, Figure 7G). Interestingly, Limosilactobacillus was significantly positively correlated with stem cell markers *Olfm4* and *Spink4*, and negatively correlated with the enterocyte marker *FABP-1* (*p* < 0.05). *Lactobacillus* showed significant positive correlations with *Spink4* and *LYZ1* (*p* < 0.05). Collectively, these results suggest that *L. mucosae* LM410 may be associated with the regulation of intestinal stem cell proliferation and differentiation and with activation of the AhR/STAT3 pathway, which may contribute to intestinal homeostasis.

## 4. Discussion

Exploring functional probiotics from stress-tolerant animals is a promising strategy for improving gut health and overall performance in livestock. The Hezuo pig is a subgroup of the plateau-type Tibetan pig breed, exhibiting strong adaptability to high-altitude hypoxic environments and notable disease resistance [35]. Elucidating the gut microbiota structure and composition of Hezuo pigs is therefore of great significance for the exploration and utilization of probiotic resources. Considering the substantial differences in rearing conditions between Hezuo and LY pig breeds, we opted not to use identical feed and housing for both, but instead selected Hezuo pig populations raised under three distinct feeding systems (semi-grazing in summer, semi-grazing in winter, and confinement in summer) and compared each of them with LY pigs. The results showed that Hezuo pigs harbor a distinct and more diverse gut microbiota, with significant enrichment of short-chain fatty acid-producing genera such as *Lachnospira, Romboutsia*, *Faecalibacterium*, *unidentified_Ruminococcaceae*, and *Subdoligranulum*. These taxa are well known for their roles in carbohydrate utilization and monoamino acid fermentation [36,37], which aligns well with the high-fiber diet and strong stress resistance of Hezuo pigs. Most strikingly, the relative abundance of *L. mucosae* was consistently and significantly higher in Hezuo pigs across all three rearing modes (TS, TW, and TS.y) compared to LY pigs, where it was nearly absent. This enrichment of fiber-degrading and SCFA-producing taxa suggests that the gut microbiota of Hezuo pigs is functionally adapted to their high-fiber, plant-based diet and may contribute to their ability to extract energy from fibrous feed sources. The persistent enrichment of *L. mucosae* across different Hezuo conditions suggests that host genetic factors may contribute to its colonization, although this remains a hypothesis requiring further investigation. Previous studies have reported that *L. mucosae* can synthesize exopolysaccharides and antimicrobial peptides [38], antagonizes pathogens, and enhances intestinal barrier function and antioxidant capacity [39,40,41]. Accordingly, we hypothesize that *L. mucosae* may represent a core functional microbiota specifically associated with the superior adaptability of Hezuo pigs, warranting future functional validation through fecal microbiota transplantation or gnotobiotic models.

To further explore this hypothesis, we successfully isolated a novel strain, *L. mucosae* LM410, from the feces of Hezuo pigs and evaluated its probiotic properties. In vitro assays revealed that this strain could effectively inhibit the growth of *C. perfringens type* C and exhibited potential strong oxygen radical scavenging capacity, indicating its possible inhibitory and antioxidant capacities. In a subsequent animal trial, oral administration of LM410 (especially at the medium dose, 1 × 10^9^ CFU/mL) significantly improved intestinal morphology, barrier integrity, immune function, and antioxidant status in weaned piglets. Specifically, VH and VCR were increased, implying enhanced absorptive surface area; serum D-LA levels were reduced, and the mRNA expression of tight junction proteins (*ZO-1*, *Occludin*, and *Claudin-1*) in the jejunum was upregulated, collectively confirming strengthened intestinal barrier function. These morphological and barrier improvements are consistent with previous reports using other probiotic strains, such as *L. plantarum* 299v in piglets [42] and *B. pumilus* 315 in goats [43]. Notably, a clear dose-dependent pattern was observed in this study, with beneficial effects at low and medium doses but detrimental effects at the high dose (1 × 10^10^ CFU/mL), including intestinal villus damage, increased serum D-LA levels, and elevated pro-inflammatory cytokines (*IL-6* and *IL-1β*). These adverse effects suggest that excessive probiotic supplementation may overstimulate the intestinal immune system or disrupt mucosal barrier function, potentially through excessive bacterial colonization or metabolic overload. This finding highlights the importance of dose optimization for practical applications: while the medium dose (1 × 10^9^ CFU/mL) appears effective and safe, the high dose should be avoided. Future studies investigating the mechanistic basis of this high-dose effect are warranted to establish safe dosage guidelines in swine production.

However, despite these positive effects on gut health, *L. mucosae* LM410 supplementation did not significantly increase final body weight or ADG in piglets. We speculate that the 14-day intervention period might have been too short to translate improved intestinal function into growth performance, as body weight gain is a cumulative process requiring longer-term modulation. This observation echoes the findings of Kang et al. [20], who reported that *L. mucosae* LM1 enhanced immunity and regulated gut microbiota in piglets without significantly affecting growth performance.

The improvements in the physical barrier were paralleled by systemic immune and antioxidant regulation. The complement system is a core effector of humoral immunity [44]. C3 and C4 are the most abundant complement molecules in serum and can directly regulate inflammatory responses, pathogen clearance, and immune processing [45,46]. In the present study, *L. mucosae* LM410 supplementation significantly enhanced humoral immunity, as evidenced by elevated serum C3 and C4 levels in the medium-dose group, and, at the medium dose, promoted an anti-inflammatory cytokine profile characterized by reduced *IL-1β* and increased *IL-10*. The effects at the medium dose are consistent with other probiotics such as *Bacillus coagulans* [47] and other *L. mucosae* strains [48]. Collectively, this dose-dependent immune modulation, anti-inflammatory at the medium dose but pro-inflammatory at the high dose, underscores the critical importance of dose selection when considering *L. mucosae* LM410 for immunomodulatory applications. However, at the high dose, the expression of pro-inflammatory cytokines *IL-6* and *IL-1β* was significantly elevated, indicating a potentially unfavorable inflammatory response. Concurrently, *L. mucosae* LM410 robustly boosted the host antioxidant defense system. All groups treated with *L. mucosae* LM410 showed significantly reduced serum MDA concentration and increased SOD, CAT, and T-AOC activities, with the medium-dose group displaying the highest catalase and total antioxidant capacity. These findings align with studies on *Lactobacillus johnsonii* RS-7 [49] and *Clostridium butyricum* in weaned piglets [50], as well as with *L. mucosae* LMU1001 in aged mice [18].

Existing studies have confirmed that *L. mucosae* can modulate the gut microbiota in pathogen-infected mouse models, but relevant research in piglets remains scarce. Our study revealed that administration of *L. mucosae* LM410 altered the gut microbial composition of piglets. The relative abundances of multiple SCFA-producing genera, including *Lactobacillus*, *Limosilactobacillus*, *Faecalibacterium*, and *Holdemanella*, were significantly increased, whereas potential pathogens such as *Streptococcus* and *Chlamydia* were suppressed. *Holdemanella* can produce the anti-inflammatory octadec-3-enoic acid [51], *Limosilactobacillus* can upregulate the expression of intestinal tight junction proteins [52], and *Faecalibacterium* helps supply energy to colonocytes and alleviate intestinal inflammation [53]. Consistent with these findings, previous studies have also reported that probiotic supplementation, including lactic acid bacteria complexes and *Enterococcus faecium*, can increase fecal *Lactobacillus* abundance in weaned pigs [54]. Furthermore, functional prediction of microbiota indicated that tyrosine metabolism and toluene degradation pathways were significantly enriched in the *L. mucosae* LM410 group. Microbial tyrosine metabolism has been increasingly recognized for its roles in immune regulation [55], oxidative stress mitigation [56], and colitis alleviation [57], while enrichment of the toluene degradation pathway suggests that this strain may reduce the accumulation of toxic metabolites in the gut [58]. Correlation analysis revealed that the relative abundances of *Lactobacillus* and *Limosilactobacillus* were significantly positively correlated with *Claudin-1* and C3, and significantly negatively correlated with D-LA, *IL-1β*, and pathways associated with bacterial motility and amino acid metabolism (e.g., flagellar assembly and bacterial chemotaxis). These findings indicate that *L. mucosae* LM410 reinforces the intestinal barrier and modulates immunity through gut microbiota-mediated tyrosine metabolism in weaned piglets.

Probiotics exert beneficial effects, in part, by producing aromatic amino acid metabolites that activate the AhR/STAT3 signaling pathway, thereby stimulating the proliferation and differentiation of ISCs [59,60,61]. ISCs are essential for repairing intestinal damage and promoting epithelial regeneration [62]. Research in piglets has shown that probiotics such as *Bacillus amyloliquefaciens* SC06 alleviate intestinal inflammation by modulating ISC proliferation and differentiation specifically via the AhR/STAT3 pathway [63]. In this study, *L. mucosae* LM410 treatment was associated with promoting the proliferation of ISCs in weaned piglets. Specifically, it upregulated the expression of ISC marker genes and promoted the differentiation of goblet cells and Paneth cells by upregulating the *Spink4* and *LYZ1* genes. Moreover, *L. mucosae* LM410 enhanced the protein expression of AhR and p-STAT3 and upregulated the gene expression of *AhR*, *CYP1B1* and *IL-22*, indicating that activation of the AhR/STAT3 pathway was associated with intestinal cell proliferation and differentiation. Aromatic amino acid metabolites, such as tyrosine, have been reported to regulate stem cell differentiation and intestinal barrier homeostasis by binding to AhR [64]. In our study, PICRUSt2-based functional prediction revealed that supplementation with *L. mucosae* LM410 was associated with upregulation of the tyrosine metabolic pathway, which positively correlated with *Limosilactobacillus* abundance and host antioxidant and barrier markers. However, because tyrosine-derived metabolites were not directly measured in the present study, and no inhibition or blocking experiments of the AhR/STAT3 pathway were performed, our data support a potential association among microbiota remodeling, tyrosine metabolism, AhR/STAT3 signaling, and ISC responses, but do not establish a causal relationship. We therefore propose the hypothesis that modulation of gut microbial tyrosine metabolism may contribute to the activation of the AhR/STAT3 pathway and the promotion of ISC proliferation, representing a potential mechanism by which *L. mucosae* LM410 supports intestinal barrier function. Future studies involving targeted metabolomics, AhR antagonists, or pathway-specific inhibitors are warranted to test this hypothesis and establish causality.

In this study, cecal contents were selected for microbiota analysis. This choice was supported by previous findings indicating that the cecum and colon are the primary sites of microbial fermentation in pigs, where short-chain fatty acids are actively produced [65]. Moreover, the cecal microbiota responds sensitively to dietary and probiotic interventions, making it an informative site for detecting microbial shifts [66]. Nevertheless, we acknowledge that the absence of colonic data limits the comprehensiveness of our evaluation. Future studies incorporating multi-site intestinal sampling are warranted to validate the regional effects of *L. mucosae* LM410.

This study is limited by the relatively small sample size. Although piglets from the same litter with uniform body weights were selected to minimize genetic variation, this approach cannot eliminate the potential bias introduced by the limited sample size. In addition, the one-pen-per-treatment design confounds treatment effects with pen effects, and piglets within the same pen are not statistically independent due to their shared rearing environment. Therefore, the present findings are exploratory and descriptive in nature. Future research should not only expand the sample size to validate our findings but also employ a design with at least three independent pens per treatment and use an in vivo model of intestinal oxidative damage to elucidate the underlying mechanisms, with a particular focus on tyrosine metabolism. This will include, but is not limited to, examining the activation or inhibition of relevant signaling pathways, screening of tyrosine metabolites, and conducting functional validation both in vitro and in vivo. Through these investigations, we aim to comprehensively elucidate how *L. mucosae* LM410 exerts its antioxidant effects, thereby providing a theoretical foundation for its practical application in healthy pig production.

## 5. Conclusions

This study found a higher abundance of *L. mucosae* in Hezuo pigs compared to LY pigs and isolated the strain *L. mucosae* LM410. Preliminary evidence suggests that supplementation with *L. mucosae* LM410 is associated with improved immunity, enhanced antioxidant defenses, and modulation of the gut microbiota. Mechanistically, our findings suggest a potential link between *L. mucosae* LM410, the predicted upregulation of tyrosine metabolism, and activation of AhR/STAT3 signaling pathways, which may be related to ISC proliferation and differentiation. Collectively, *L. mucosae* LM410 exhibits promising probiotic potential for alleviating intestinal oxidative stress and maintaining intestinal homeostasis.

## Data Availability

The 16S rRNA sequencing data generated in this study have been deposited in the NCBI Sequence Read Archive (SRA) database and are publicly available under accession numbers PRJNA1414209 (fecal samples) and PRJNA1405475 (cecal content samples). The complete genome sequence of *L. mucosae* LM410 has been submitted to GenBank under accession number PRJNA1414803. All other data supporting the findings of this study are included within the article and its Supplementary Materials and are also available from the corresponding author upon reasonable request.

## Supplementary Materials

The following supporting information can be downloaded at https://www.mdpi.com/article/10.3390/antiox15101264/s1: Supplementary Figure S1: Isolation, identification and biological characteristics of *L. mucosae* LM410. Supplementary Figure S2: The rarefaction curve of each sample. Supplementary Figure S3: PCoA based on Bray–Curtis distances and histogram of LEfSe analysis at species level. Supplementary Table S1: Nutrient composition of the diet (%). Supplementary Table S2: Primer sequences used for real-time PCR. Supplementary Table S3: Identification of isolated strains with HBI biochemistry characterization. Supplementary Table S4: Acid resistance of *L. mucosae* LM410. Supplementary Table S5: Cholesterol tolerance of *L. mucosae* LM410. Supplementary Table S6: Effect of *L. mucosae* LM410 on growth performance in weaned piglets.

## Abbreviations

AhR/STAT3 signaling pathway	Aryl hydrocarbon receptor/signal transducer and activator of transcription 3 signaling pathway
SCFA	Short-chain fatty acid
ROS	Reactive oxygen species
ISC	Intestinal stem cell
Lgr5+	Leucine-rich repeat-containing G-protein coupled receptor 5
ADG	Average daily gain
DPBS	Dulbecco’s Phosphate-Buffered Saline
LB	Luria–Bertani
MRS	De Man, Rogosa and Sharpe
OTUs	Operational taxonomic units
ASVs	Amplicon sequence variants
PCA	Principal component analysis
PCoA	Principal coordinates analysis
NMDS	Non-metric multi-dimensional scaling
KEGG	Kyoto Encyclopedia of Genes and Genomes
D-LA	D-lactic acid
C3	Complement 3
C4	Complement 4
T-AOC	Total antioxidant capacity
SOD	Superoxide dismutase
CAT	Catalase
MDA	Malondialdehyde
RT-qPCR	Real-time quantitative PCR
CPC	*Clostridium perfringens* type C
BSA	Bovine serum albumin
SDS-PAGE	Sodium dodecyl sulfate–polyacrylamide gel electrophoresis
PVDF	Polyvinylidene difluoride
HRP	Horseradish peroxidase
