# Supplementary material for: Limosilactobacillus mucosae LM410 Enhances Intestinal Barrier and Antioxidant Capacity in Weaned Piglets via Microbial Tyrosine Metabolism and AhR/STAT3 Pathway
Source: Antioxidants (Basel). 2026 Oct 1;15(10):1264. doi: 10.3390/antiox15101264 (PMC13700798; doi:10.3390/antiox15101264)
Supplement: Supplementary file 1 [file antioxidants-15-01264-s001.zip › antioxidants-4423407-supplementary.pdf]

Supplementary Information

Supplementary Figure S1: Isolation, identification and biological characteristics of *L. mucosae* LM410.

Supplementary Figure S2: The rarefaction curve of each sample.

Supplementary Figure S3: PCoA based on Bray–Curtis distances and histogram of LEfSe analysis at species level.

Supplementary Table S1: Nutrient composition of the diet (%).

Supplementary Table S2: Primer sequences used for real-time PCR.

Supplementary Table S3: Identification of isolated strains with HBI biochemistry characterization.

Supplementary Table S4: Acid resistance of *L. mucosae* LM410.

Supplementary Table S5: Cholesterol tolerance of *L. mucosae* LM410.

Supplementary Table S6: Effect of *L. mucosae* LM410 on the growth performance in weaned piglets.

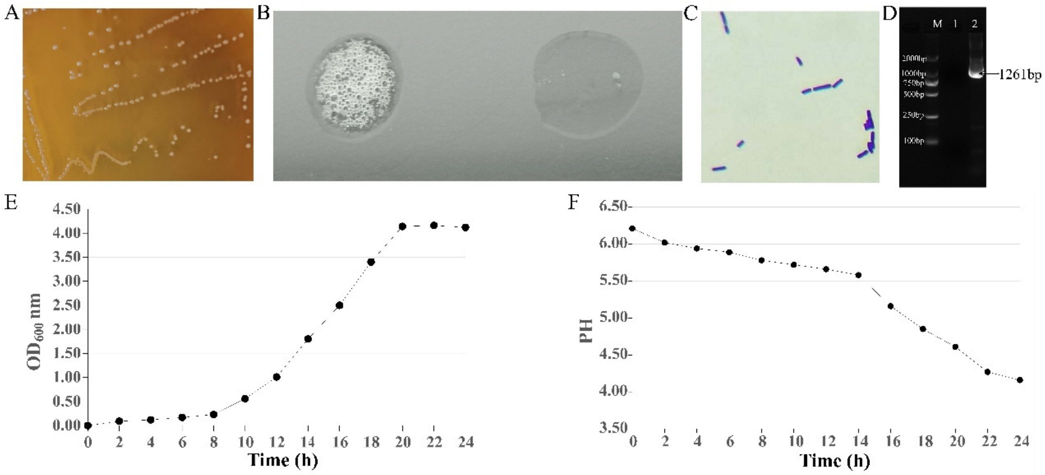

Figure S1. Isolation, identification and biological characteristics of *L. mucosae* LM410. (**A**) Colony

morphology for the isolated strains. (**B**) Hydrogen peroxide (catalase) assay for the isolated strains. (**C**) Gram-staining microscopy for the isolates (1000×). (**D**) PCR-based identification from candidate strains. M: 2000 bp marker; Lane 1: Negative control; Lane 2: Isolate candidate strain. (**E**) Growth kinetics. (**F**) Acidogenesis profile. n = 3.

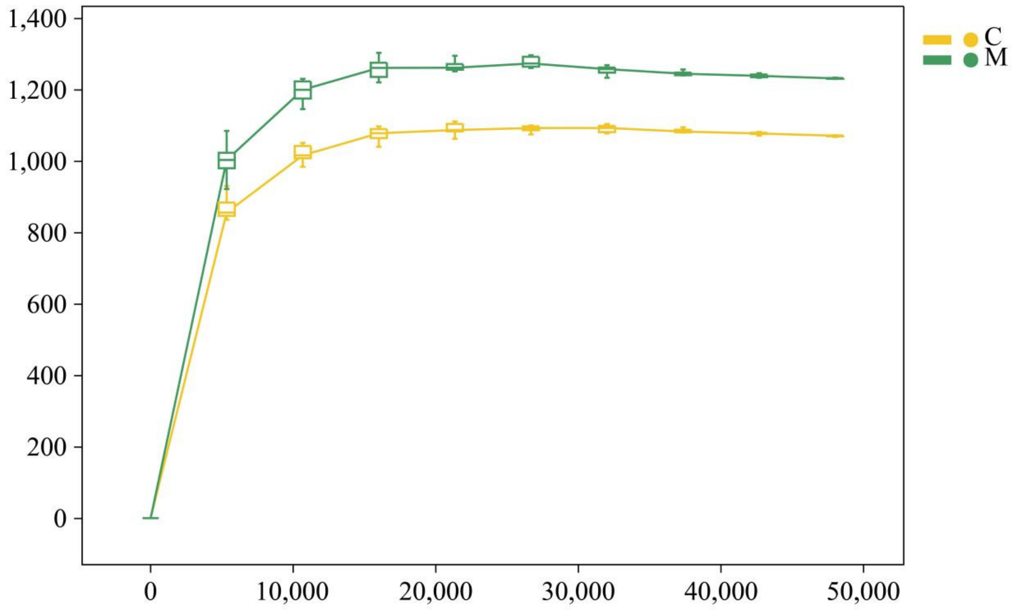

Figure S2. The rarefaction curve of each sample. n = 3.

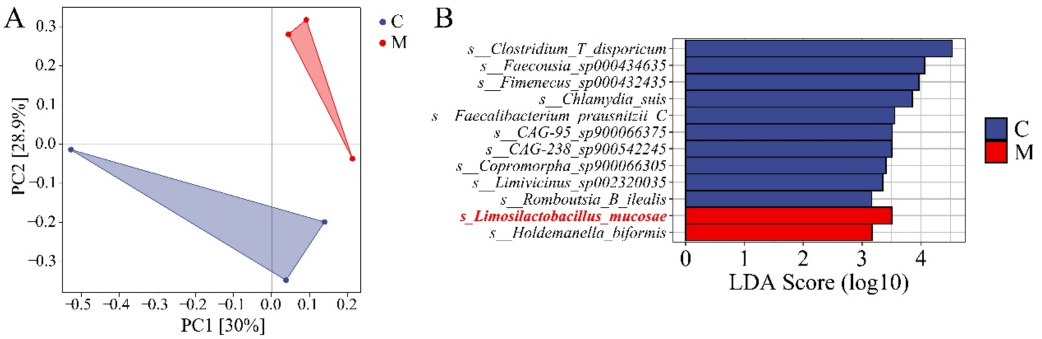

Figure S3. (**A**) PCoA based on Bray-Curtis distances. (**B**) Histogram of LEfSe analysis at species level. (Kruskal–Wallis test $P < 0.05$ and LDA score (log10) > 3.0); n = 3.

Table S1. Nutrient composition of the diet (%).

Ingredient	Content (%)
Crude protein	≥18.5
Crude ash	≤8.0
Crude fiber	≤4.1
Total phosphorus	≥0.3
Lysine	≥1.3
Calcium	0.5-1.1
Sodium chloride	0.3-0.9
Moisture	≤14.0

Table S2. Primer sequences used for real-time PCR.

Gene	Primer Sequence (5′-3′)	Length	Reference Serial Number
IL-10	F: CGGCGCTGTCATCAATTTCT<br>R: GGCTTTGTAGACACCCCTCTC	102	NM_214041.1
IL-6	F: TCTGGTGATGGCTACTGCCT<br>R: CCGGAGAGGTGAAGAGCATTT	95	NM_214399.1
IL-1β	F: CAAGAGGATACTTCCCCTGACC<br>R: CTTCCTTGGCAGGTTCAGGTA	150	NM_001302388.2
Occludin	F: CAGTGGTAACTTGGAGGCGT<br>R: CCGTCGTGTAGTCTGTCTCG	104	NM_001163647.2
Claudin-1	F: GGACTAATAGCCATCTTTGT<br>R: CAGCCATCCGCATCTTCT	88	NM_001244539.1
ZO-1	F: TGAGTTTGATAGTGGCGTTG<br>R: TGGGAGGATGCTGTTGTC	298	XM_021098827.1
GAPDH	F: AGTATGATTCCACCCACGGC<br>R: TACGTAGCACCAGCATCACC	139	NM_001206359.1
Lgr5	F: AAGCCTTTGTAGGCAACCCT<br>R: GAGGCACCATTCAAAGTCAGTG	124	NM_001315762.1
Olfm4	F: TCACGGAGGTGTGTGGAATG<br>R: CTGGGGATGTCCTGCCAAAT	96	XM_003482903.4
Sox-9	F: GAAGCTGGCGGATCAGTACC<br>R: GCCGTTCTTCACCGACTTTC	193	NM_213843.3
Spink4	F: CATTGTGGACAGAGAAGTGCC<br>R: TGGGTCGTATATCCGGGAGC	109	NM_213864.1
MUC2	F: GGACGACACCATCTACCTCAC<br>R: AGGCCAGCTCGGGAATAGA	132	XM_021082584.1
LYZ1	F: GCGCTTCTCCTCCTTTCTGT<br>R: CCTATAGCCGTCCATGCCAG	96	NM_214392.2
FABP-1	F: CGGAAATCGTGCAGAATGGG	93	NM_001004046.2

	R: CACACTCCTCTCCCAAGGTG		
KRT20	F: CACAGTGAGCACGGAAGAGT<br>R: CTCTGGTAGGTGCGTCTGTG	69	NM_001444899.1
Villin1	F: CTAGACAGGCTCATCCACCA<br>R: CCTGGCCGATCCAGTAATGA	224	NM_001444847.1
AhR	F: CCAACATCACCTACGCCAGT<br>R: CAGTTTTCTGCACGGGCTTC	51	NM_001303026.1
CYP1B1	F: AGCTCCTCTGTTCCGCTTTC<br>R: ATCATTGGCAGGTCAGTGGG	67	XM_021087620.1
IL-22	F: GGCCCTGAAGAATGAATGCAC<br>R: ACCTTGACTGTGAAGTTTGGC	145	XM_021091968.1

Table S3. Identification of isolated strains with HBI biochemistry characterization.

Items	Results
Aescin	+
Fibrosaccharide	-
Maltose	+
Mannitol	-
Salicin	-
Sorbitol	-
Sucrose	+
Candyfloss	+
Inulin	/
Lactose	+
1% Sodium hippurate	/

Note: + positive; - negative; / not performed.

Table S4. Acid resistance of *L.mucosae* LM410.

pH Value	Viable Count	Survival Rate (%)
6.20	9.32	100.00
4.00	8.26	88.63
3.00	7.88	84.55
2.00	7.78	83.48

Table S5. Cholesterol tolerance of *L. mucosae* LM410.

Added Amount of Pig Bile Salt (%)	Viable Count (lgCFU/mL)	Survival Rate (%)
0.00	9.32	100.00
0.10	8.92	95.70
0.20	8.84	94.85
0.30	8.73	93.67

Table S6. Effect of *L. mucosae* LM410 on growth performance in weaned piglets.

Items	Treatment			
	C	L	M	H
Initial weight (kg)	7.81 ± 0.72	8.02 ± 0.81	8.30 ± 0.93	7.18 ± 0.29
Final weight (kg)	10.17 ± 0.98	10.20 ± 1.01	10.53 ± 0.86	9.50 ± 0.36
ADG (g/d)	157.11 ± 20.06	145.11 ± 37.37	149.11 ± 12.76	154.44 ± 43.00

Note: C, pigs fed the basal diet without LM410; L, pigs fed the basal diet with 1×10^8 CFU/mL LM410; M, pigs fed the basal diet with 1×10^9 CFU/mL LM410; H, pigs fed the basal diet with 1×10^{10} CFU/mL LM410. $n = 3$. Data are presented as the mean ± standard deviation (SD).
